# Developments in the field of health policy analysis in low- and middle-income countries: a review of published literature 2008–2023

**DOI:** 10.1093/heapol/czag079

**Published:** 2026-06-25

**Authors:** Eleanor Beth Whyle, Lucy Gilson, Zubin Cyrus Shroff

**Affiliations:** School of Public Health, Health Policy and Systems Division, University of Cape Town, South Africa; School of Public Health, Health Policy and Systems Division, University of Cape Town, South Africa; Department of Global Health and Development, London School of Hygiene and Tropical Medicine, Keppel Street, London WC1E 7HT, United Kingdom; Alliance for Health Policy and Systems Research, World Health Organization, 20 avenue Appia, 1211 Geneva, Switzerland

**Keywords:** health policy, policy processes, policy analysis

## Abstract

This review maps the development of health policy analysis (HPA) in low- and middle-income countries (LMICs) since 2008, when Gilson and Raphaely (Gilson L, Raphaely N. The terrain of health policy analysis in low and middle income countries: a review of published literature 1994–2007. *Health Policy Plan* 2008;23:294–307. https://doi.org/10.1093/heapol/czn019) published a review clarifying the state of the field and tracing its evolution. Subsequent years have seen some progress in capacity strengthening for HPA in LMICs, but the extent of development of the field has not been formally assessed since that time. We used a combination of bibliometric, thematic, and narrative analysis to better understand the nature of the research being undertaken, identify weaknesses in the evidence base, and gauge maturation of the field. Despite the relatively restrictive focus of this review, we identified 629 articles reporting empirical studies of LMIC policy experiences, revealing substantial growth in the volume of published HPA since 2008. However, significant imbalances in knowledge production remain, with low-income country experiences, and those of North Africa and Central Asia still neglected, and with the dominance of HIC authors persisting despite efforts to strengthen the field in LMICs. At the same time, the review finds evidence of maturation, reflected in deeper theoretical engagement, greater methodological diversity, the use of comparative approaches to enable causal explanation and cross-context learning, and the emergence of robust bodies of work on specific health policy issues. Taken together, the findings point to a field that has grown in scale, scope and analytic sophistication, but still requires deliberate nurturing. Purposeful efforts by researchers, funders, and institutions are needed to ensure that HPA realizes its full potential and contributes to progressive policy change. Improving domestic and regional funding, supporting LMIC institutions and researchers, deepening theoretical engagement, and encouraging analytical HPA that enables explanation and cross-context learning are essential to sustaining the development of the field and ensuring that it continues to generate rigorous, relevant insights and contributes to progressive policy change.

Key messages

- The field of health policy analysis (HPA) in low- and middle-income countries (LMICs) has expanded markedly since 2008, with a substantial increase in published work. However, persistent imbalances remain: authors based in high-income countries continue to dominate, and studies of policy processes in low-income settings, and North Africa and Central Asia remain limited.
- The field shows clear maturation, reflected in a wider range of methods and deeper theoretical engagement. These developments are particularly evident in implementation research, where social science theory is increasingly used to illuminate how power, trust, and values shape policy processes.
- Methodological diversity has also grown, with more studies using analytical and comparative approaches that allow for causal explanation and the generation of lessons of wider relevance. These approaches mark an important shift from descriptive to explanatory analysis, strengthening the field’s ability to support learning across contexts.
- Sustaining progress requires deliberate and coordinated effort. Deepened theoretical engagement, equitable authorship and partnership practices, domestic and regional investment in LMIC institutions, and strengthened prospective and policy-engaged HPA are essential to ensuring the field continues to inform, and help shape, progressive policy change.

## Introduction

Academic research into social policy, including health policy, was initiated in the USA and Europe in the mid-20th century, and research on policy processes is now an established academic field in these settings. In contrast, health policy research in low- and middle-income countries (LMICs) is a more recent area of work and has often focused on policy content specifically (Walt and Gilson 1994, Gilson and Raphaely 2008, Walt 2018). Nonetheless, since the 1990s, the study of health policy processes, or health policy analysis (HPA), has undergone substantial development in LMICs with a focus on understanding the social factors, political context, and power dynamics (Walt 1994, Walt and Gilson 1994, Reich 1995) that help explain ‘who made what policy decisions, when, why and how, and with what consequences’ (Gilson et al. 2018, p.10).

In 2008, Gilson and Raphaely published a systematic narrative review of literature on health policy processes in LMICs from 1994 to 2007. This review was intended to clarify the state of the field and trace its evolution by mapping ‘what analysis has been undertaken of health policy processes in LMICs, and by whom?’ (Gilson and Raphaely 2008, p. 295). The review revealed a relatively small body of empirical HPA evidence, spread thinly over a range of topics and geographies, and found the field was dominated by ‘Northern-based’ authors. The review authors concluded that HPA in LMICs ‘remains in its infancy’ (p. 295), pointing to weaknesses within the knowledge base, including limited contextualization; limited use of analytical methods that allow for the explication of the socially constructed nature of policy (such as discourse and framing analysis); a preponderance of purely descriptive or broadly explanatory (rather than analytical) articles; and limited use of policy analysis theory to deepen analysis, allow for explanation (as opposed to only description), and support the development of generalizable lessons (Gilson and Raphaely 2008). They also noted a scarcity of studies undertaken with the intention of directly influencing contemporary policy change processes. Accordingly, Gilson and Raphaely called for efforts to deepen and extend HPA for LMICs. They recommended dedicated capacity strengthening, more purposive programmes of HPA work seeking to generate evidence to support policy change (such as multi-country studies and sets of studies conducted in series to build understanding), more engagement between health policy analysts and policy-makers deliberately seeking to influence policy change, and greater use of theory to explain policy change and explore causality.

Subsequent years have seen some progress in capacity strengthening for HPA in LMICs—such as the publication of a HPA Reader (Gilson et al. 2018) and an HPA Fellowship programme to support doctoral-level LMIC students doing HPA work in LMICs (Gilson et al. 2021). However, the extent to which the field of HPA in LMICs has developed over time has not been considered since the 2008 review. This review, therefore, maps the terrain of HPA in LMICs between 2008 and 2023, where possible, considering the findings against those of the earlier review. By combining bibliometric analysis with more in-depth review of selected papers, we seek to support the further growth of the field by better understanding the nature of the research being undertaken, identifying persistent weaknesses in the evidence-base, and developing methodological insights to encourage rigorous, innovative, and high-quality HPA.

## Materials and methods

While the present review is, in some ways, a follow-up to the 2008 review, a somewhat different methodological approach was taken—as explained below, with more details provided in Supplementary material.

### Data collection and search strategy development

Given that HPA is a multi-disciplinary field, sharing ‘fuzzy’ boundaries with the wider field of health policy and systems research (HPSR), as well as health economics, implementation sciences, and policy-focused social science, the development of a search strategy for a field review is a complex and necessarily iterative endeavour (Gilson 2012, Swinkels 2020). From the outset, it was acknowledged that many HPA-relevant items might not be explicitly identified as ‘health policy analysis’. As a result, it was necessary to develop a broad and inclusive search strategy and use title and abstract screening to eliminate articles without a policy process focus.

We developed this search strategy for PubMed, beginning with a focused search to develop a set of obviously relevant items both to inform the final search strategy and serve as a quality check for future searches. The focused search combined two search strings to identify articles using the term ‘health policy analysis’ and discussing LMIC contexts:

1. ‘health policy analysis’
2. LMIC synonyms and country names

**Figure 1:**
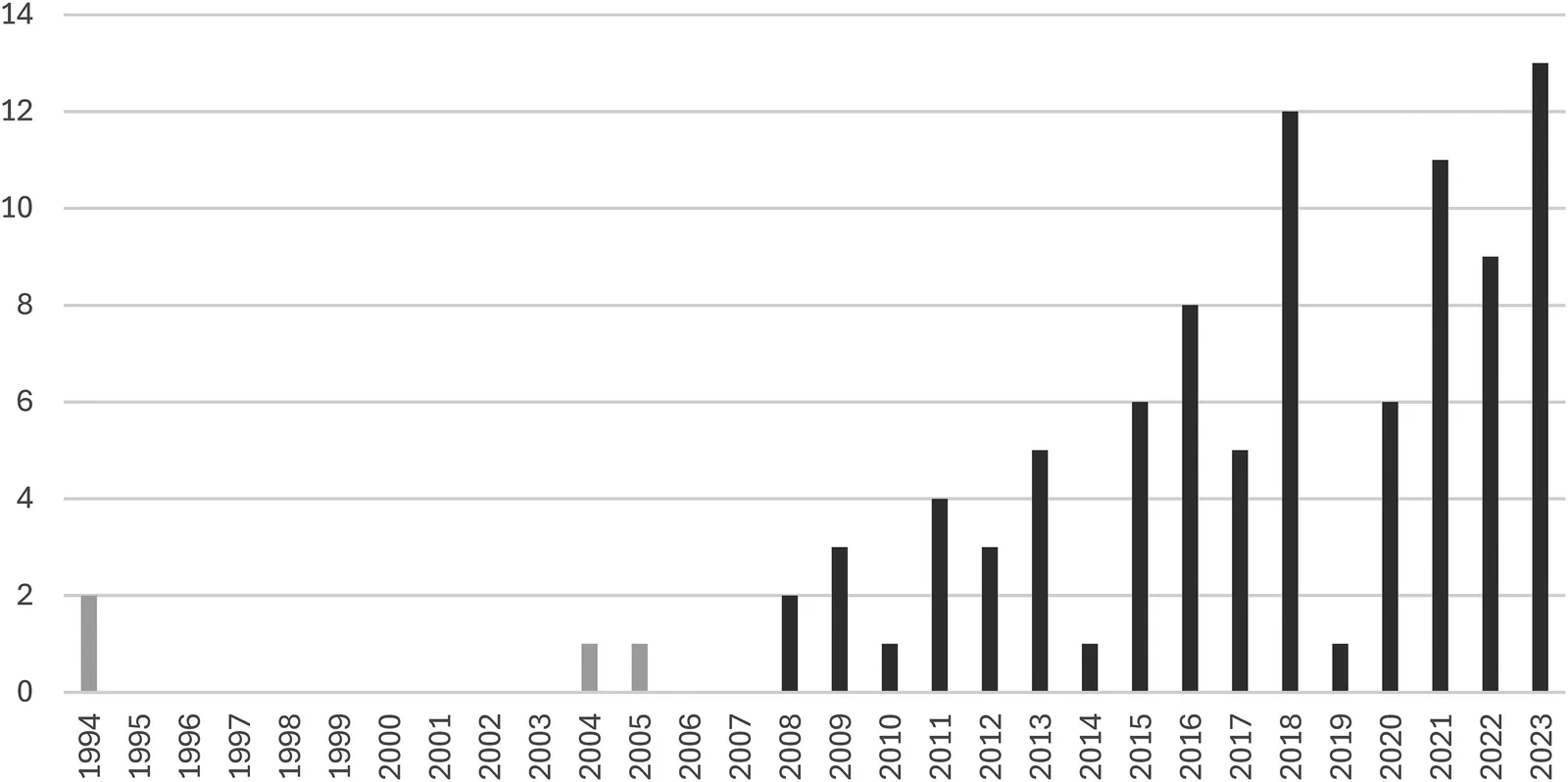
Results of a purposive search in the PubMed database showing the increase in literature since 2008.

This yielded 94 items of which 74 were deemed relevant. This search also clearly indicated the effect of the Gilson and Raphaely review on the HPA evidence-base in LMICs, as seen in Fig. 1.

Drawing from this initial set of 74 items, we then developed a more inclusive search strategy, to capture the full range of HPA-relevant material, that combined five strings:

1. ‘Health’ in title/abstract
2. ‘Policy’ or ‘policies’ in title/abstract.
3. Policy process term in title/abstract. These policy process terms were developed in consultation with a health sciences librarian based on the 2008 Gilson and Raphaely review and other key HPA papers identified through the preliminary focused search. See Supplementary Data S1 for details.
4. LMIC country names in title/abstract. (A decision was taken to exclude synonyms for LMIC—such as ‘developing country’, ‘emerging economy’, or ‘third world’—as the inclusion of these terms resulted in many items that had a general focus and did not explore health policy process(/es) in any particular setting(/s).)
5. MeSH major topic. The list of relevant MeSH topics and headings was developed iteratively based on the MeSH indexing of the 74 items identified as relevant. See Supplementary Data S1 for details.

**Figure 2:**
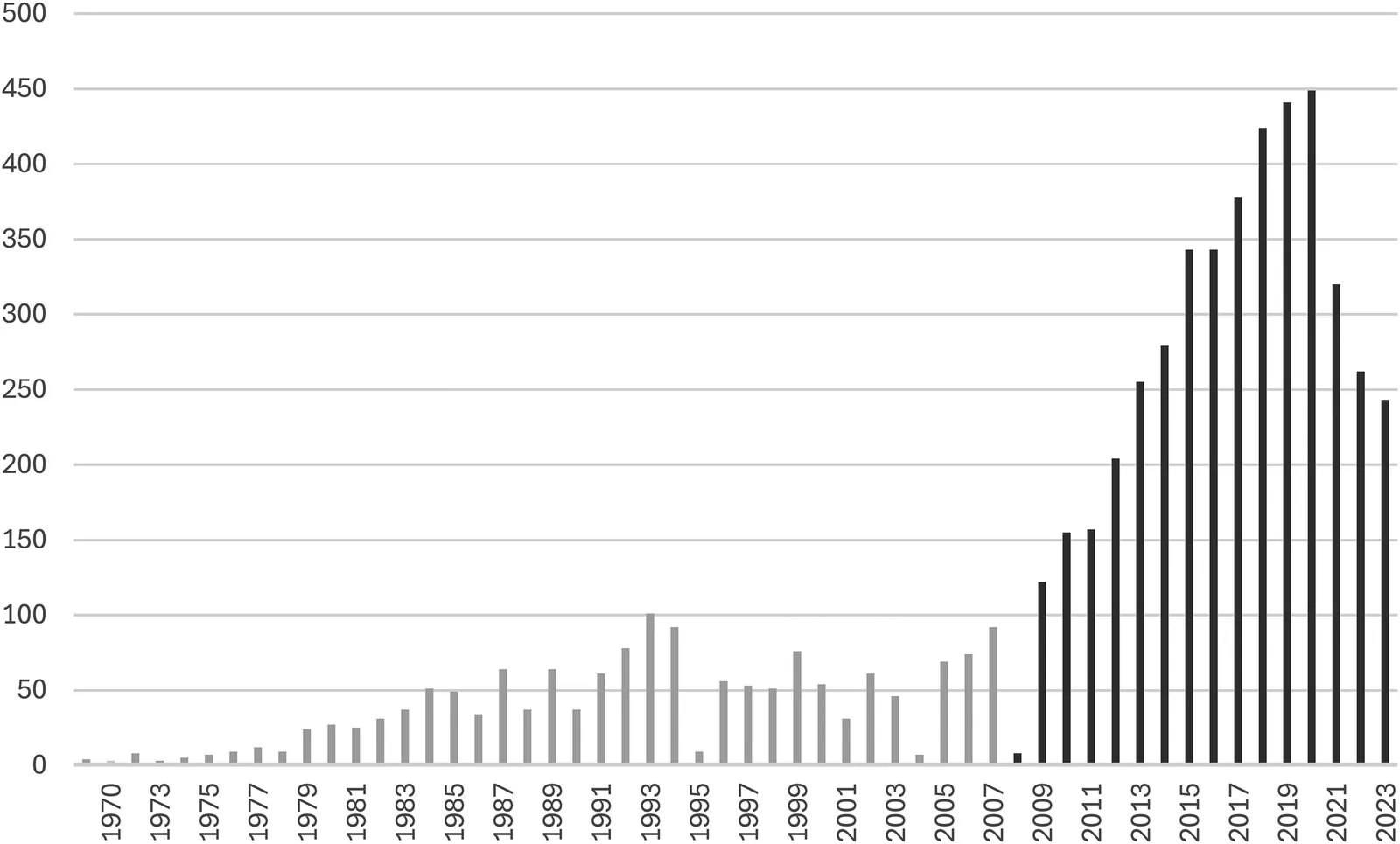
Results of more inclusive PubMed search showing increase in literature since 2008.

Limited to English and 2008–23, this search yielded 3 988 items and, checking against the initial 74 items, we determined it had achieved an optimal balance of sensitivity and specificity. This search also confirmed the increase in literature since 2008, as seen in Fig. 2. All 3 988 items were downloaded to EndNote for screening.

This methodological approach has limitations that must be recognized. Searching academic databases only precludes the identification of, for example, relevant book chapters. We recognize that this, together with the choice of databases, may have skewed our findings to some extent (because, for example, social science on health policy might be more likely to be published in books or in discipline-specific journals not indexed in the included databases).

The broad and inclusive search in PubMed was then supplemented with purposive searches in AfricaWide and SciELO to ensure the full range of African and Latin America and the Caribbean (LAC) evidence was captured (see Supplementary Data S1 for details). In AfricaWide, our search yielded 639 items, of which 191 were deemed relevant and downloaded to EndNote. In SciELO, these searches identified 348 items, of which only 55 were in English and published since 2008. This suggests that there is a significant amount of HPA-relevant evidence in LAC languages other than English. All 55 were deemed relevant and downloaded to EndNote. In total across the three databases and after the removal of duplicates, 4147 items were downloaded into an EndNote library.

### Screening

**Figure 3:**
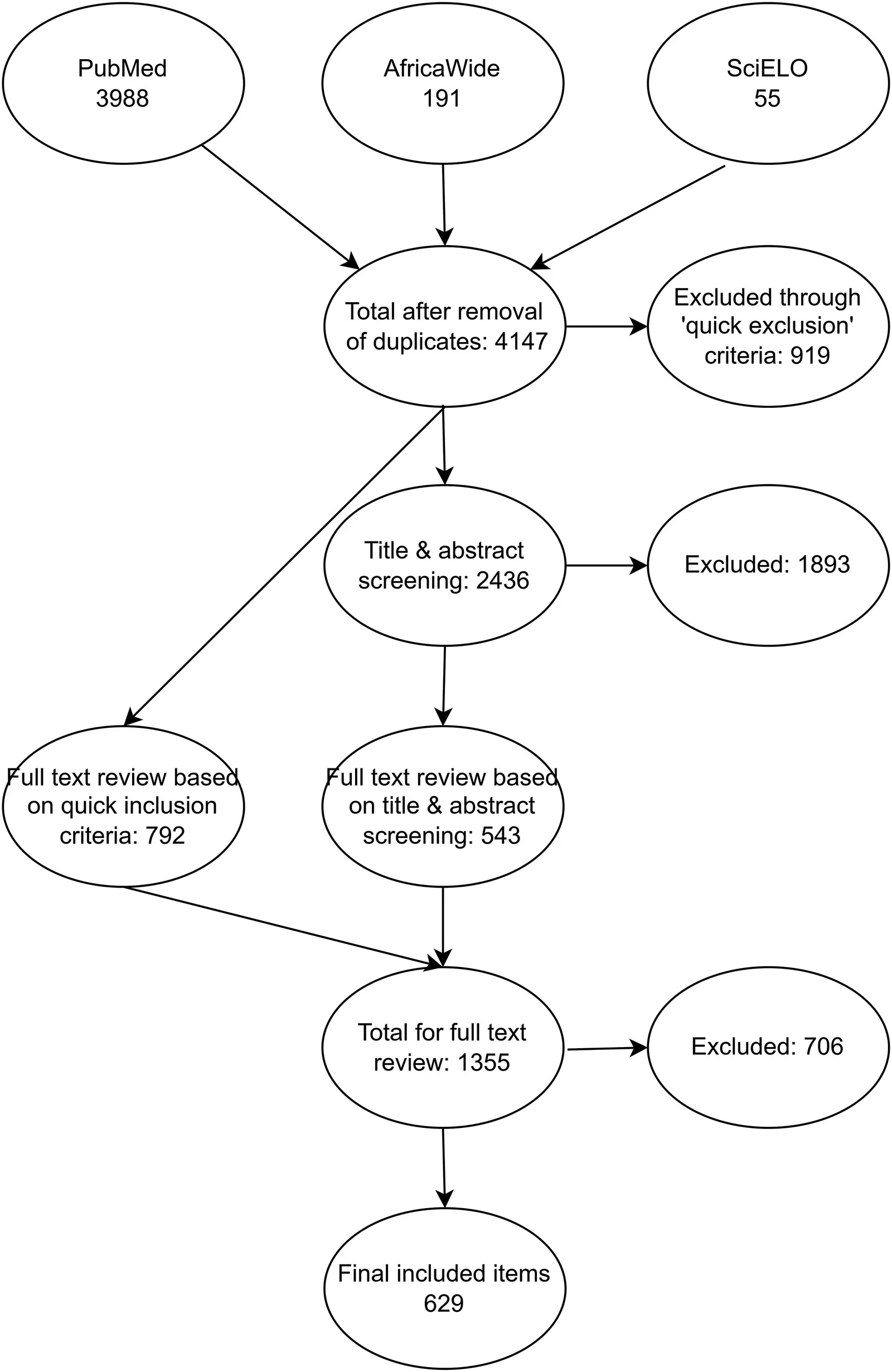
Flow diagram of study selection process.

Figure 3 presents the screening process diagrammatically. The screening process was developed collaboratively by the first and second authors. The first author conducted the screening, and borderline cases were discussed by all authors. To facilitate screening, we began with a process of ‘quick inclusions’ and ‘quick exclusions’ by downloading the full texts for all 4147 items and using EndNote’s search function to search the full texts for phrases that indicate the item is likely relevant and should be included for full text review, or likely irrelevant and should be screened for exclusion (see Supplementary Data S1 for ‘quick inclusion’ and ‘quick exclusion’ criteria). Seven hundred and ninety-two items were included for full text review after this initial screening.

Because the present review is focused on empirical studies of HPA experiences at national or sub-national levels, we sought to exclude the following:

- Literature reviews studying some aspect of policy or policy-making in general, without consideration of any particular policy experience
- Policy impact assessments or evaluations rather than process analyses
- Studies of policy-makers’ or users’ perceptions or attitudes, without considering process
- Papers focusing on producing or translating evidence for policy, i.e. to make policy recommendations (without connecting these to an analysis of the policy process)
- Studies of policy content only, without considering process
- Commentaries and editorials
- Purely technical/quantitative analyses (including technical health economics papers)
- Study protocols

After excluding 919 items based on our ‘quick exclusion’ criteria, we used title and abstract screening to screen the remaining 2436 articles and excluded 1893 items, leaving 1355 items for full text review (including the 792 items identified for full text review through the ‘quick inclusion’ criteria). After full text review, we excluded an additional 706 items. In addition to the exclusion categories listed earlier, common reasons for exclusion were:

- Paper simply describes a policy, programme, and/or policy problem without an analytical component or attention to process, or is a descriptive report of experience without reporting methods (purely descriptive studies of policy process were included as long as data collection and analysis methods were reported).
- Focus is general—no analysis of specific policy process or country context (this category includes reviews).
- Focus is on mechanism for public participation.
- Focus is on a service delivery or health system issue without consideration of a process of policy change.

Ultimately, we retained 629 items for review.

### Data analysis

Our data analysis approach combined bibliometric analysis, evidence mapping, and elements of thematic and narrative synthesis to determine the amount of literature produced in various geographical and thematic areas and identify gaps and weaknesses. The bibliometric analysis was conducted by EBW with support from LG and ZCS. The qualitative analysis was conducted by EBW and LG with support from ZCS.

The bibliometric analysis was conducted by exporting the reference data (including abstracts) from EndNote to Excel and extracting data for each reference on the year of publication, publication journal, authorship, the institutional affiliation of the authors, and the country(/ies) of focus of the study. This analysis also included a light-touch thematic analysis: by reading the abstract of each article, we extracted data on the health or health system topic, HPA theme or phase of focus, HPA theory or framework applied (if any), study design, and data analysis methods.

During this process, we also identified particularly illustrative HPA articles, understood as articles displaying one or more of the characteristics of HPA called for in prior HPA reviews, including Walt and Gilson (1994), Gilson and Raphaely (2008), and Walt *et al*. (2008). Accordingly, articles flagged were those that had one or more of the following characteristics:

- Engages with and accounts for the influence of intangible factors such as power, politics, process, discourse, and values
- Compares across cases or contexts to explain change and/or draw out lessons or insights of wider relevance
- Applies HPA or social science theory to move from description to explanation
- Centres actors and their interests and the contested nature of policy change
- Locates policy change experiences in rich descriptions of context (including historical)
- Is an analysis conducted in order to influence policy change

Because our data collection methods differed from those of the prior review, we compare our included papers with the narrower set of 164 empirical examples of health policy change experiences in LMICs identified by Gilson and Raphaely.

## Findings

In what follows, we present an evidence map based on the results of the bibliometric and thematic analyses. We begin with evidence of the growth of the field and then present an analysis of the geographic distribution of studies and authors. Finally, we provide a synthesis of literature in terms of the policy phases considered, methods and study designs employed, theories and frameworks used, and subject areas and topics covered (or neglected), comparing our findings with those of the earlier review wherever possible. Throughout, we draw attention to particular papers or groups of papers to illustrate the extent of development of the field and the value of HPA concepts and methods to explaining and contributing to policy change. We recognize that there are many excellent examples of scholarly HPA, only a small selection of which can be featured here.

### Growth in the field

**Figure 4:**
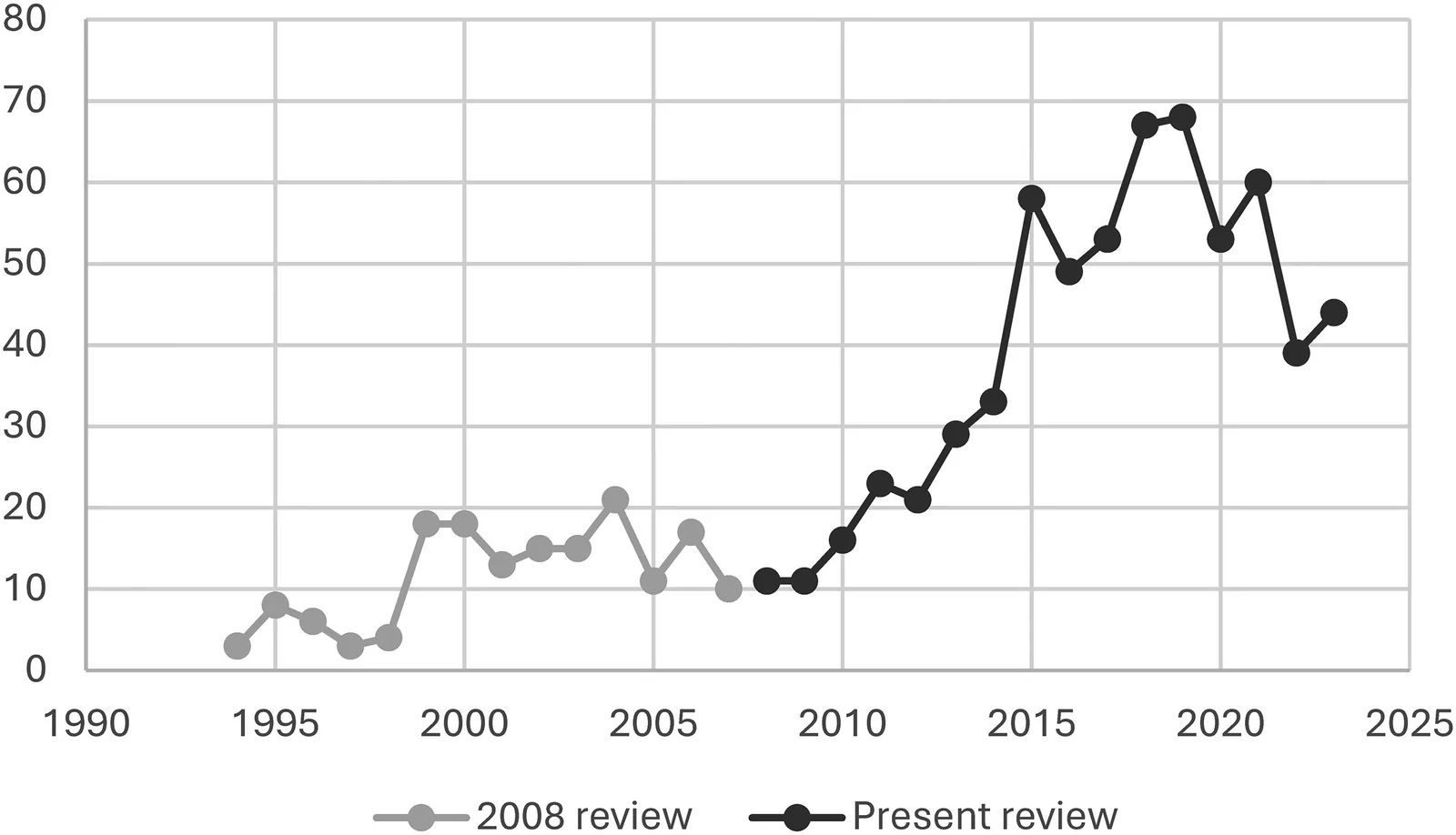
Number of included articles per year for 2008 review and present review.

Despite the relatively restrictive focus of this review, we identified 629 articles published over 15 years (2008–23). Accounting for the methodological differences between this and the 2008 review, our findings nevertheless suggest significant growth in the field over time. The 629 articles included in the present review amount to approximately 40 articles per year, compared to the ±12 articles per year identified in the 2008 review. Figure 4, showing the number of included articles by year for both reviews, also indicates a significant (and relatively steady) acceleration in the number of HPA articles being produced since 2008.

### Geographic distribution of studies, publications and authors

In this section, we present an analysis of the geographic distribution of the country of focus of the included studies and the institutional base of the study authors.

#### Country of focus

To assess the geographic distribution of the evidence-base, we analysed the included material by country of focus. Across all 629 included studies, we identified studies on 92 LMICs. Table 1 presents the countries that were the focus of 10 or more articles. The full list of countries, along with the number of studies identified for each, is presented in Supplementary Data S2. South Africa was the focus of the highest number of articles [61 of 886, 6.9%], followed by India (49, 5.5%), Ghana (44, 5%), and Brazil (40, 4.5%). Note that 886 is higher than the number of included articles because some articles studied experiences in more than one country.

**Table 1:** The countries that were the focus of more than 10 included articles and the number of articles per country.

Country of focus	No. of articles
South Africa	61
India	49
Ghana	44
Brazil	40
Iran	40
Kenya	39
Uganda	37
Nigeria	33
China	32
Tanzania	26
Malawi	25
Ethiopia	23
Thailand	23
Burkina Faso	22
Vietnam	20
Mexico	19
Indonesia	17
Nepal	14
Cambodia	13
Cameroon	13
Zambia	13
Bangladesh	12
Philippines	12
Pakistan	11

**Figure 5:**
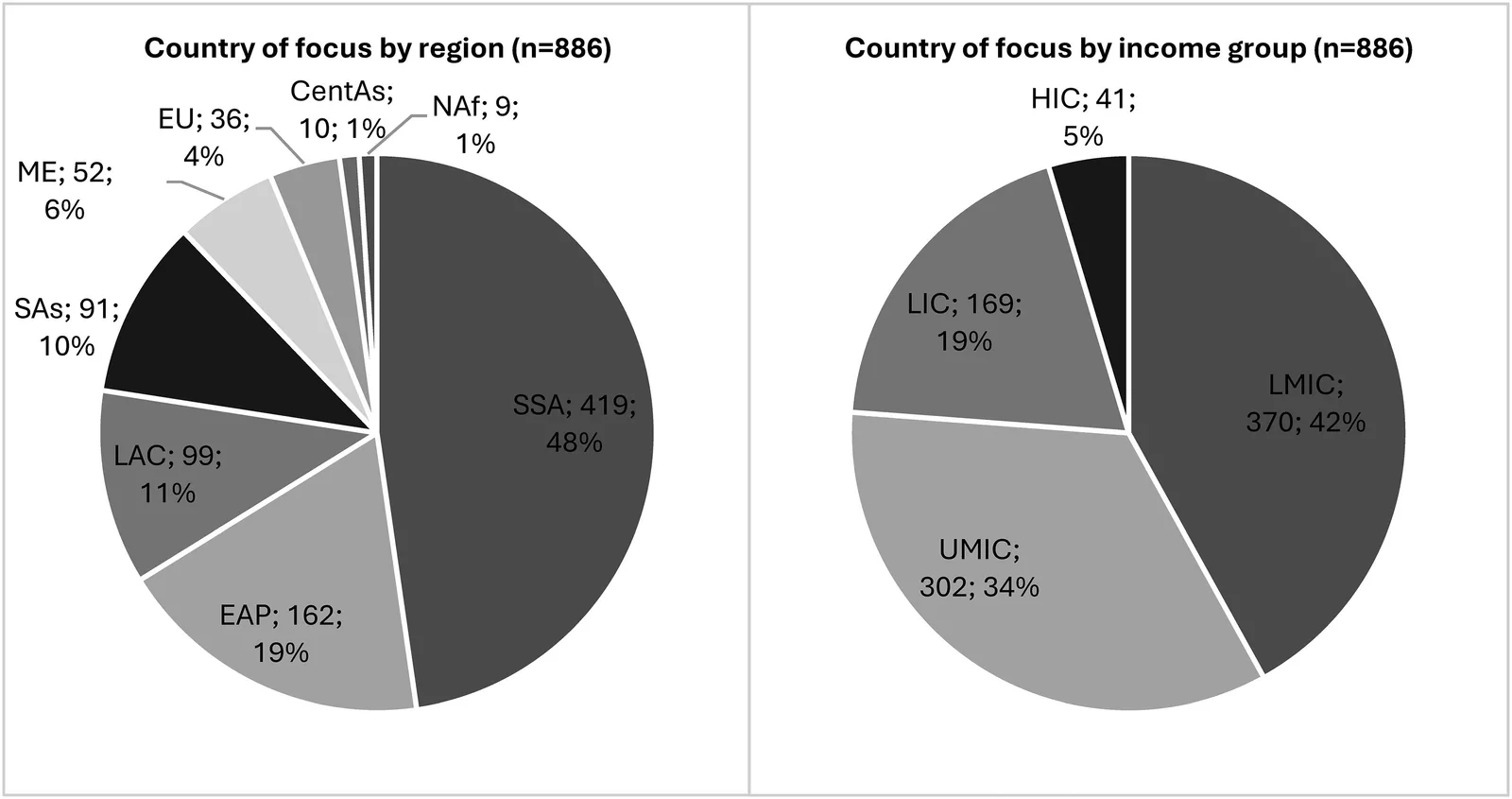
Country of focus by region and income group. SSA, sub-Saharan Africa; EAP, East Asia and Pacific; LAC, Latin America and the Caribbean; SAs, South Asia; ME, Middle East; EU, Europe; CentAs, Central Asia; NAf, North Africa; LMIC, lower-middle income country; UMIC, upper-middle income country; LIC, low-income country; HIC, high-income country.

Figure 5 presents the country of focus of the included articles by World Bank income group and region. Note that there are HIC countries included here because papers covering a number of countries were included if one or more of the countries of focus were LMICs. Almost half (48%) of all included articles focused on a country (or countries) in SSA, while North Africa and Central Asia were the focus of only 9 and 10 articles respectively, or 1% of included papers, each. While the LAC region accounted for 11% of included studies, Brazil and Mexico alone accounted for 60% of articles on LAC countries, with very few articles on other countries in the region. With respect to income groups, lower-middle-income countries were the focus of 42% of all included papers, followed by upper-middle-income countries at 34%. Only 19% of articles focused on low-income countries (LICs). While LIC experiences are still under-studied and some regions remain particularly neglected, these findings indicate progress has been made since 2008, when Gilson and Raphaely did not identify any papers focusing on LICs, or on North African, Central Asian, or Mediterranean country experiences, and found that almost half (47%) the articles focused on UMIC country experiences.

It is also noteworthy that most studies examine the experience of only one country. Only 95 (15%) of articles considered more than one country—either considering a particular topic across a region (22, 3%) or income group (4, >1%) or analysing two or more comparator countries (69, 11%). The value of cross-country comparisons (and comparative approaches in general) in HPA is discussed further below.

#### Journal of publication

The vast majority (92%) of included articles were published in international journals. Table 2 presents the journals in which ten or more articles were published. The most common journal by far was *Health Policy and Planning*, which is likely a reflection of the Journal’s particular focus on LMIC contexts, and the fact that it has a section dedicated to health policy processes. The remaining 8% of articles were published in regional or national journals, such as the Brazilian Journal *Ciência & Saúde Coletiva* (in which we identified 11 included articles) and the *Pan African Medical Journal* (in which we identified 4 included articles). Gilson and Raphaely found a high concentration of articles in core health policy journals, with 53% of articles in *Health Policy and Planning* and *Social Science and Medicine*. We found slightly less concentration in core journals with 45% of articles published in journals devoted to health policy specifically, suggesting perhaps, that there has been some progress in integrating the field of HPA into the wider literature or demonstrating the relevance of HPA work to a wider audience.

**Table 2:** The journals in which 10 or more articles were published and the percentage of the total number of included articles per journal.

Journal	Number of articles	% total
Health Policy and Planning	140	22.3%
Health Research Policy and Systems	61	9.7%
BMC Health Services Research	35	5.6%
International Journal of Health Policy and Management	32	5.1%
BMC Public Health	29	4.6%
International Journal of Equity in Health	24	3.8%
Social Science and Medicine	18	2.9%
Global Health	17	2.7%
Health Policy	13	2.1%
International Journal of Health Planning and Management	13	2.1%
BMJ Global Health	12	1.9%
PLOS One	12	1.9%
International Journal of Health Politics, Policy and Law	11	1.7%
Global Health Action	10	1.6%
Global Public Health	10	1.6%

#### Authorship and authors’ institutional affiliation

To better understand where HPSR knowledge is being produced, and by whom, we also analysed the authors of the included articles along with the institutional affiliation of the authors (at time of publication). There were 2 221 unique authors. However, 83% of authors contributed to only one article, and 86% of first authors first authored only one article. While this may suggest a relatively open and diverse field, it may also indicate that some researchers with an interest in health policy are not able to sustain engagement in the field.

**Figure 6:**
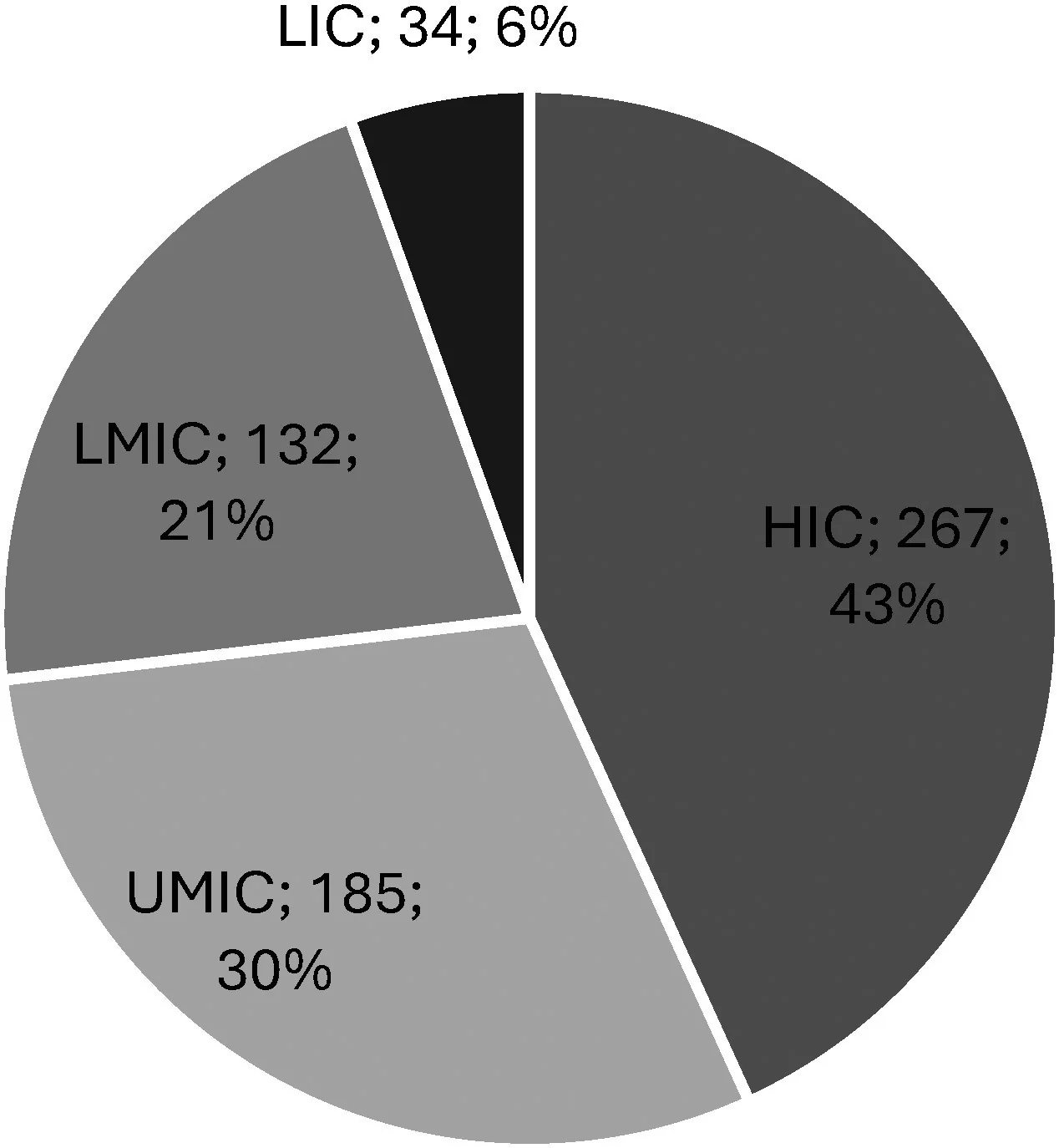
First author institutional affiliation by region (n=618*). HIC, high-income country; UMIC, upper-middle income country; LMIC, lower-middle income country; LIC, low-income country. *For a few included items it was not possible to identify the institutional affiliation of the first author. Thus, the number of first authors in this analysis is slightly lower than the total number of included papers.

Analysing the institutional affiliation of first authors reveals that 43% of first authors were based at institutions in HICs and only 27% at lower-middle or LIC institutions (see Fig. 6), despite the fact that we only included articles with an LMIC country focus. Furthermore, while 19% of included articles focused on LICs, only 6% of first authors were based at LIC institutions. Of course, this is a fairly crude analysis—accounting only for authors’ institutional affiliation at the time of publication, not necessarily their ‘home’ or country of origin. We recognize that many authors’ institutional affiliations may not fully reflect their nationality, experience, or expertise. Nonetheless, these findings show that HIC institutions and authors continue to dominate the field, reflecting a persistent and problematic maldistribution of power in knowledge production.

**Figure 7:**
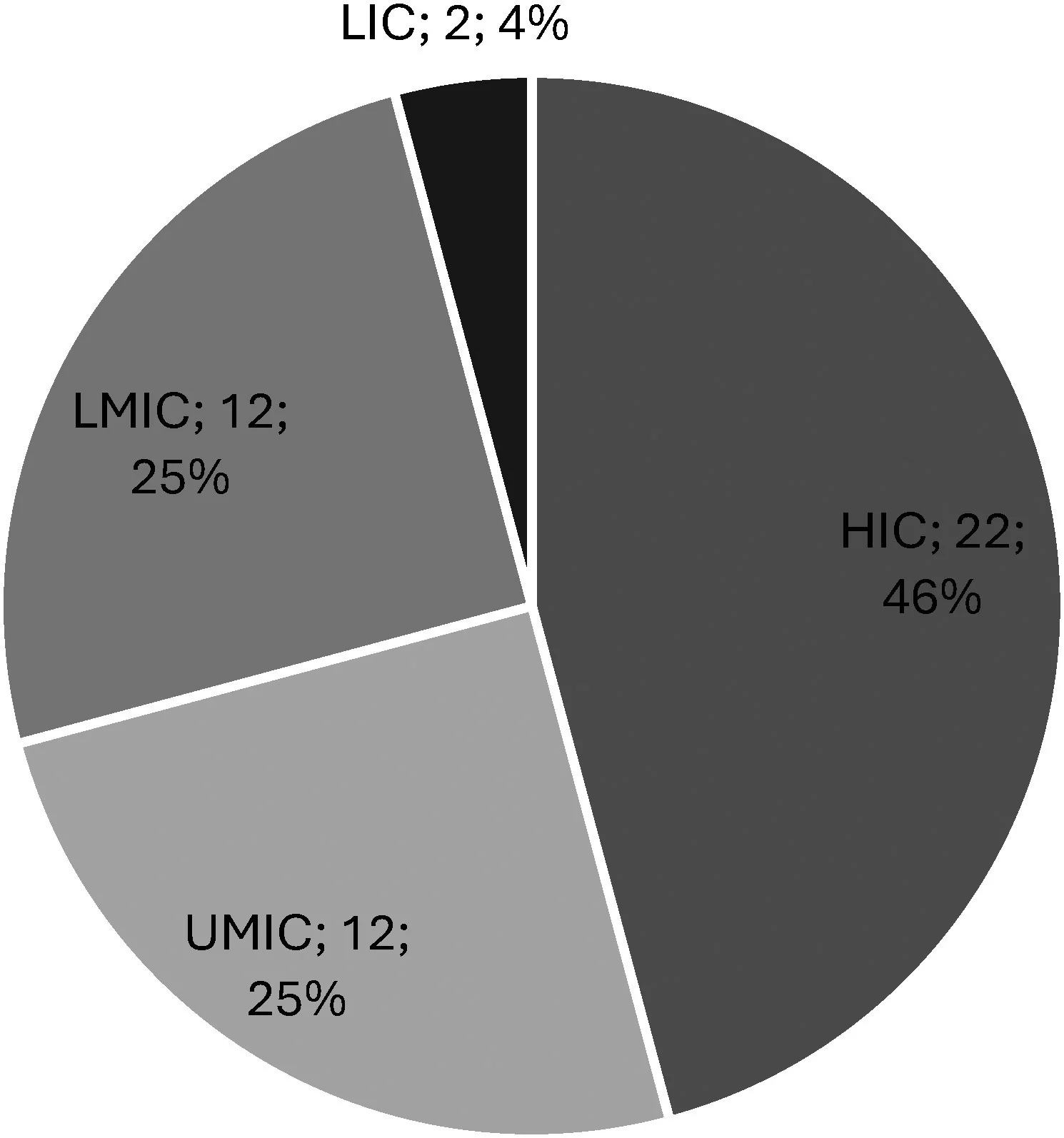
Highly recurrent authors’ institutional affiliation by income group. HIC, high-income country; UMIC, upper-middle income country; LMIC, lower-middle income country; LIC, low-income country.

**Figure 8:**
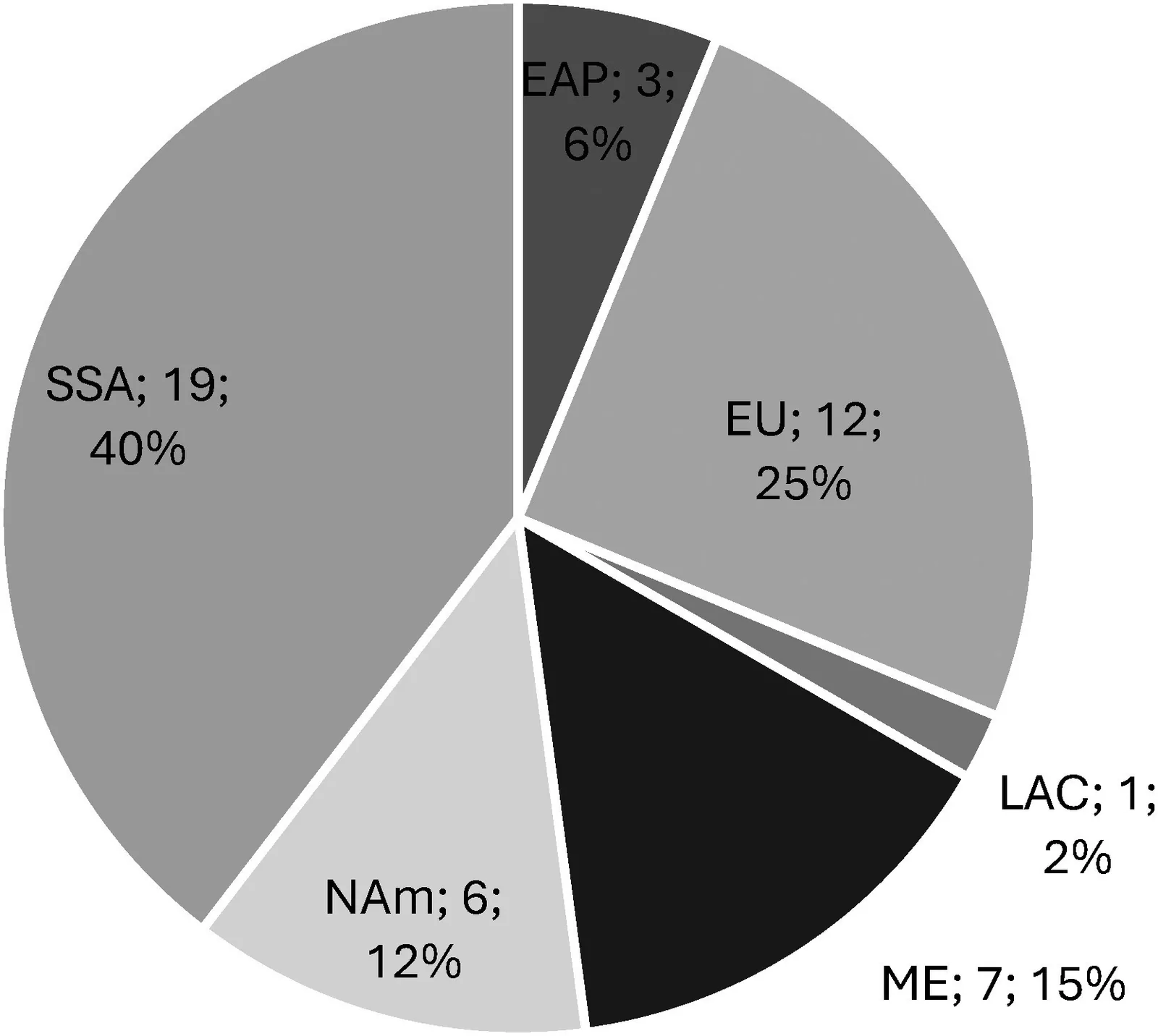
Highly recurrent authors’ institutional affiliation by region. SSA, sub-Saharan Africa; EAP, East Asia and Pacific; EU, Europe; LAC, Latin America and the Caribbean; ME, Middle East; NAm, North America.

These trends are mirrored in an analysis of highly recurrent authors. Forty-nine authors (2.2%) contributed to five or more included articles. Analysing these highly recurrent authors’ institutional affiliations according to the World Bank country and lending groups reveals an unsurprising concentration in HICs, with 46% of highly recurrent authors (authors with 5 or more articles) being based at a HIC institution (Fig. 7). Figure 8, which presents highly recurrent authors’ institutional affiliation by region, reveals an under-representation of authors based in LAC countries, Central Asia, North Africa, and South Asia. Analysis of first authors reveals a similar pattern: of the 71 individuals who have first-authored two or more articles, almost half (46%) were affiliated with institutions in HICs, and only two were based in institutions in LICs at the time of publication. Similarly, Gilson and Raphaely found that of the 33 individuals that first-authored two or more articles, only 21% were based at ‘Southern’ institutions.

Overall, the analysis of authorship trends may suggest that LMIC researchers face barriers in finding a local or regional institutional base willing and able to support their work in the field of HPA and highlights the need to continue to strengthen institutional capacity to support HPA in LMICs, guard against ‘parachute research’, and break down systems of knowledge production that are not founded on deep knowledge of local social, political, and cultural realities and that are likely to be extractive.

### Health policy analysis phases and themes

**Figure 9:**
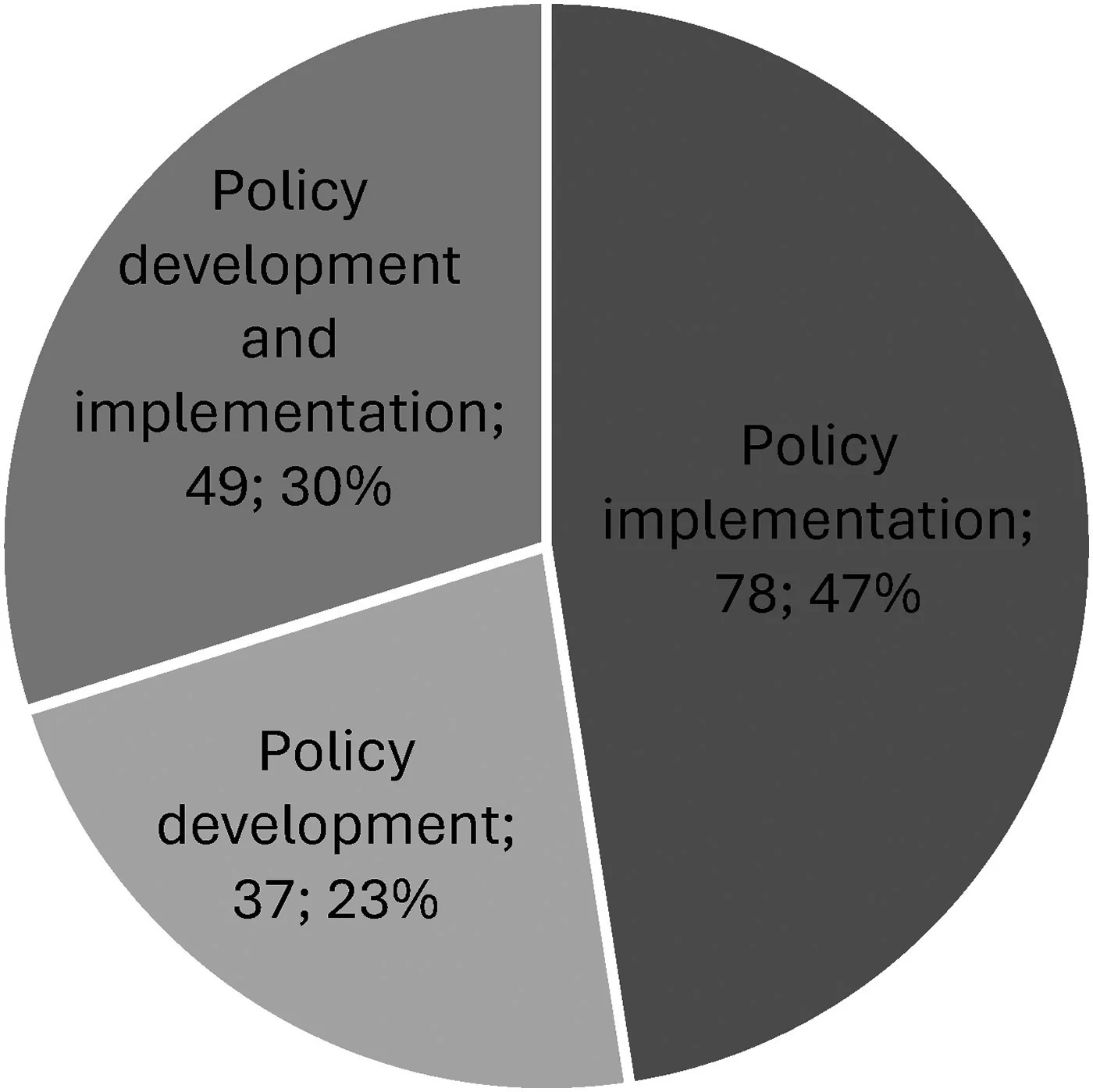
No. of articles by stage of policy process for 2008 review.

**Figure 10:**
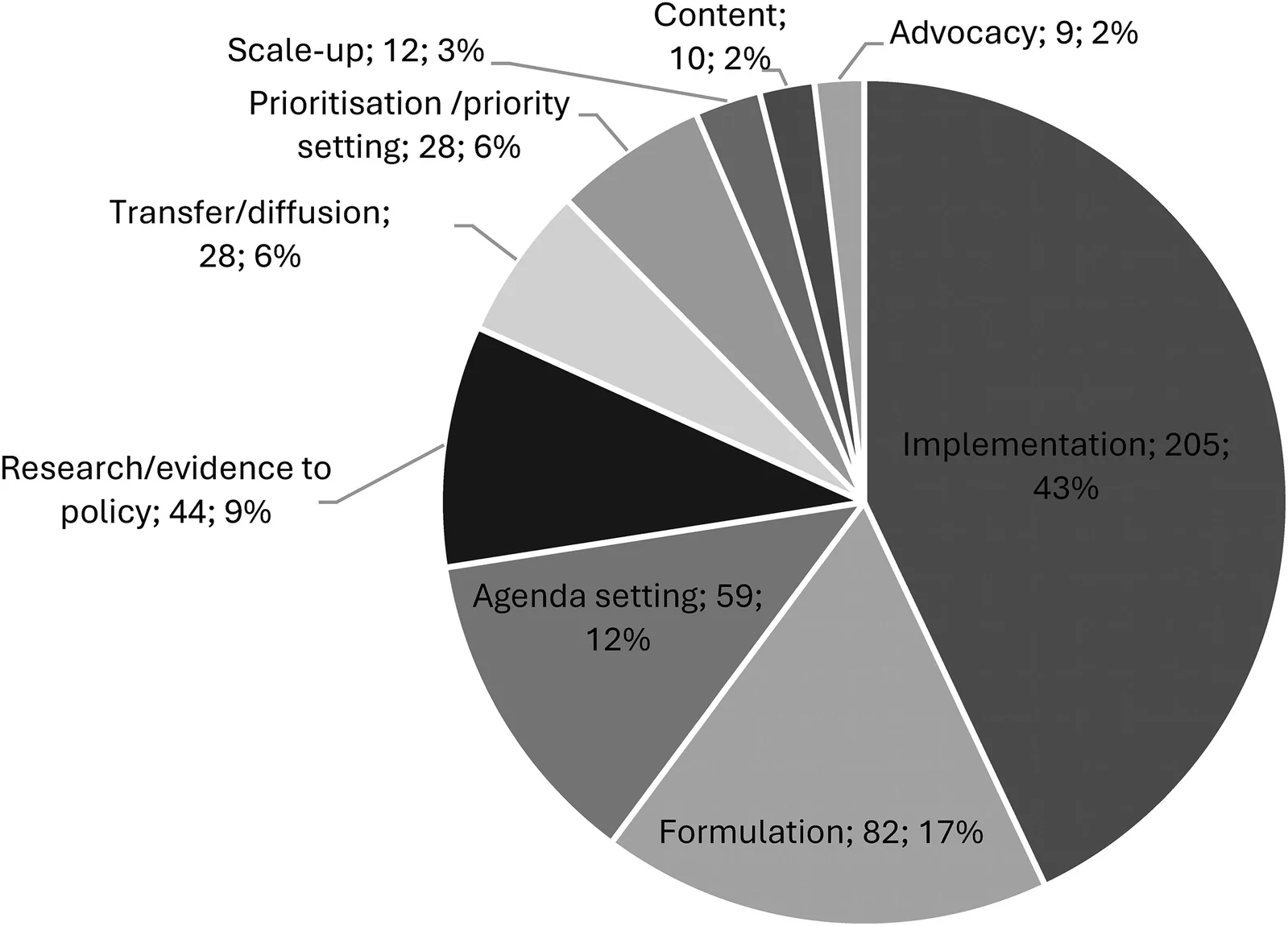
No. of articles by HPA phase or theme (present review).

In this section, we survey the HPA phases and themes covered in the evidence-base. The 2008 review categorized articles according to the policy phase of focus as either policy development-focused, policy implementation-focused, or both, finding that most articles were implementation-focused (see Fig. 9). In the present study, for the vast majority of articles (±70%), it was possible to categorize the policy phase of focus more specifically than in the earlier review. We categorized articles using the following phases, recognizing when articles covered more than one phase: agenda-setting, transfer/diffusion, prioritization/priority-setting, research/evidence to policy, formulation, implementation, scale-up, and advocacy. We also noted where the analysis was focused on policy content rather than a phase of the policy process. This in itself suggests significant development in the field, since many more authors are now using HPA language to locate their study in the policy process. Figure 10 shows the range of HPA phases or themes covered in the included articles for the present study and the number of articles in each category. One hundred and ninety-two articles (30%) were classified as ‘general’ as they concerned the policy process as a whole. Implementation-focused studies comprised the largest group (43%), followed by policy formulation (17%) and agenda-setting (12%). There were also a significant number of articles on the influence or role of evidence and research in policy change processes, the transfer or diffusion of policy ideas between contexts and prioritization of particular health and health system issues and concerns. Our study excluded policy evaluations that did not account for issues of politics and process, but we note a dearth of empirical evidence on the politics of evaluation.

Where it was not possible to classify an article by policy phase, it was usually because the article explores the policy processes more generally, without restricting the analysis to any particular phase or phases. In addition, however, some papers do not use classic policy theory ‘language’. Commonly, authors use ambiguous terms such as ‘adoption’ or ‘introduction’—the term ‘adoption’, for example, is used to refer either to the uptake of a global policy at the national level (which we, following Gilson and Raphaely, categorized as policy transfer – global to national), or the implementation or scale-up of a home-grown policy.

#### Health policy analysis on implementation

In both the present and previous review, studies focused on implementation accounted for almost half of all included papers. This is an important finding in a field in which there is a persistent concern that political analysis of implementation remains relatively neglected (Gilson et al. 2018, Campos and Reich 2019, Buse et al. 2024). In fact, in this study, we identified a number of examples of particularly strong and methodologically innovative implementation-focused health policy analyses. As a set, this group of papers exposes both the value of using concepts from the broader field of HPSR to understand how policy processes unfold within, and are influenced by, complex adaptive systems (see Box 1 for a brief explication), and of drawing on social science theory in HPA.

Box 1 **Studies of implementation processes recognizing the intersection between policy processes and complex adaptive health systems**

The body of included implementation papers reveals a number of studies drawing on concepts and theories from the wider field of health systems analysis, to better understand how policy implementation processes play out within complex health systems. Several papers locate the implementation process within complex adaptive systems without explicitly applying theories or conceptual frameworks from HPSR. For example:

- Gómez (2015) explains Brazil and USA’s different responses to the obesity epidemic in terms of the causal contribution of system-level factors such as the fragmented and competitive organization of public health institutions in USA and Brazil’s established PHC institutions combined with a long history of social health movements and their values being incorporated into national legislation.
- Islam *et al*. (2018) account for the influence of changing context at the systems level on the implementation of a policy to contract-out services for urban primary care in Bangladesh.
- Erasmus *et al*.’s (2017) paper exploring the implementation of two equity-focused policies in two district hospitals in South Africa is similarly systems-oriented, albeit focusing at a more micro-level, with the authors highlighting the causal contribution of organizational factors, including organizational culture and organizational trust.

The review also reveals a few papers that draw more explicitly on CAS theory in implementation analysis. These include the following:

- Nigenda *et al*.’s (2016) analysis of the implementation of Mexico’s innovative Seguro Popular de Salud health financing policy using a CAS framework to understand the system dynamics that result in adaptation and modification of the policy
- Ogbuabor *et al*.’s (2019) analysis of the implementation of the Free Maternal and Child Healthcare Program in Nigeria from a CAS perspective

Taken together, these papers demonstrate the analytical value of recognizing the influence of systems dynamics on policy processes and the potential of using existing frameworks for HPSR to do so.

Regarding the latter, the review identified a number of implementation-focused papers drawing on concepts and theories from social and political sciences to rigorously account for the influence of what the HPSR literature refers to as ‘software factors’—particularly power and values—in implementation (Sheikh et al. 2011). For example, Lehmann and Gilson (2013) draw on VeneKlasen and Miller’s typology of power dimensions and Long’s actor interface analysis approach to explore practices of power in the implementation of a community health worker programme in South Africa. The analysis helps explain how a multi-faceted set of policy goals were ‘thinned down and subverted’ by policy implementers, resulting in a *reduction* in available community health worker services, while also demonstrating that actors can use their discretionary power to support policy implementation. This study is also noteworthy in that it recognizes the significance of actors' affective responses to policy change. Parashar *et al*. (2020) also use Long’s theory to analyse practices of power in the implementation of the *Janani Shishu Suraksha Karyakram* policy for free maternal and child health services in India, arguing that the theory reveals ‘the often neglected and lived realities’ (p. ii74) of implementers and explains implementation challenges rooted in power asymmetries.

In a similar vein, Salve *et al*. (2018) use concepts from Bourdieu’s theory of practice—particularly ‘field’ (a relational space where contestation plays out) and ‘capital’ (the resources and assets that individuals bring to relational contestation)—to understand the social dynamics of implementation of a public-private mix TB policy in India. The authors found that some implementation challenges were a product of implementing actors feeling poorly treated, and, as a result, seeking to retain their position in the ‘field’ in ways that hampered collaboration.

Sheikh and Porter (2011) also use an interpretivist analytical approach, drawing on post-positivist social science theories of power, to unearth how front-line doctors are able to resist and subvert policy guidelines on HIV testing. The authors explain the ‘implementation gap’ in terms of doctors’ relationship with hospital administrators (who were seen as protectors rather than enforcers of policy) but also in terms of the ways in which the policy process intellectually disempowers doctors by excluding their concerns and beliefs from decision-making fora, resulting in ‘frequent conflicts between the requirements of HIV testing policies, and doctors’ own values and appreciations of situations’ (p. 88).

Finally, misalignment between policies and providers' values is further highlighted in Aniteye and Mayhew’s (2013) analysis of implementation failures of Ghana’s liberal abortion law. Using Lipsky’s street-level bureaucracy (SLB) theory, the authors expose how providers balance the tension between their personal beliefs, the stigma associated with providing abortion services, and their duty to care, by using their discretion to offer services or not, or adopting coping mechanisms such as not referring for care, giving vague referrals (so that it’s up to the patient whether they manage to receive care or not), or providing clandestine services. The paper also assesses the applicability of SLB theory to the case, noting that provider values are as important as resource constraints in determining behaviour and explaining implementation failure, and provider discretion is often significantly constrained by organizational hierarchies, with negative consequences for implementation. Both Gilson and Raphaely (2008) and Erasmus *et al*. (2014) note SLB as particularly widely applied in HPA, and the present analysis demonstrates the ways in which the theory is being extended, including through integration with other social and political science ideas.

#### Prospective health policy analysis and health policy analysis on advocacy as neglected areas in health policy analysis

This review demonstrates that there is still very little HPA on processes seeking to bring about policy change—either analyses of policy advocacy processes or examples of prospective HPA (in the sense of analyses to support policy change rather than prospective methods). Gilson and Raphaely similarly identified very few articles examining experiences of efforts to influence policy change, and in their recent review, Paina et al. (2024) confirm that prospective HPA remains in its infancy, particularly in LMICs. The few examples that were evident in the present review—which we briefly outline here—demonstrate the complexity of this work and the value of using policy analysis to better understand the processes by which researchers and advocates seek to contribute to policy change.

##### Health policy analysis studies of advocacy processes

While the review revealed very few studies of advocacy processes, the small body of work in this area suggests there is significant value in better understanding policy advocacy processes. Okeke *et al*. (2021) use Sabatier and Jenkins-Smith’s Advocacy Coalition Framework to explore how advocacy groups worked to generate and sustain political priority for maternal and child health in Nigeria after the abrupt cessation of a social protection scheme. The study reveals how ‘loose…alliances…of committed individuals and institutional policy actors with dense interorganizational and interpersonal ties’ can bring about policy change based on shared beliefs and common goals. Similarly, Michaud-Létourneau *et al*. (2019) present a real-time evaluation of the advocacy efforts undertaken to translate the 1981 World Health Assembly code on marketing of breast-milk substitutes into national legislation in nine countries. The authors identify 15 critical tasks undertaken by advocacy organizations that helped bring about legislative change, many of which highlight the importance of understanding the legislative and political context, and pre-empting opposition from powerful stakeholders. Finally, Pelletier *et al*. (2013) use case studies of three advocacy processes for nutrition policy—in Uganda, Vietnam, and Bangladesh—to draw out lessons on the practice of advocacy, including the importance of informal meetings, credibility (and the involvement of actors widely regarded as credible), and social mobilization. However, the authors also note the need for further, more detailed, studies of advocacy processes. While these specific studies demonstrate the value of HPA for advocacy processes, the review suggests the field of study remains inchoate.

##### Prospective uses of health policy analysis

We also identified three studies that demonstrate the value of prospective HPA for bringing about policy change. While not strictly empirical, Gilson *et al*.’s (2012) paper drawing on experiences of stakeholder analysis for UHC reforms in Tanzania and South Africa develops important insights on how HPA, particularly HPA using stakeholder analysis methods, can be used to support policy change. In both cases, analysts were able to develop alternative UHC reform proposals that accounted for the degree of support or opposition they would likely garner from powerful stakeholders. Although, as the authors note, lessons about stakeholder responses to reforms are complex, contextually contingent, and likely not generalizable, the paper generates insights into how to use stakeholder analysis techniques and the value of those techniques for policy analysts seeking to influence policy change processes.

Two further studies present empirical prospective analyses *for* policy change. Surjadjaja and Mayhew (2011) conducted a prospective case study of policy debates on abortion legalization in Indonesia—using Kingdon’s three streams model, the HPA triangle framework, and stakeholder mapping tools to establish whether there was a window of opportunity for policy change. Their finding, borne out by subsequent events, was that there was an opportunity to pass a Bill, provided pro-reformists were able to work with other stakeholders, including the media, to turn public and parliamentary opinion in favour of the Bill. The authors concluded that policy analysis theory and tools can inform effective strategies for policy change, but note that the efficacy of such academic work requires collaboration with change advocates and blurring the boundaries between analysts and practitioners.

Mukanu *et al*. (2023)’s prospective policy analysis of nutrition policy reforms in Zambia, using interviews and document review informed by Kingdon’s multiple streams theory, is a final excellent example of the potential of prospective policy analysis. The authors found that while malnutrition was recognized as a serious issue, adolescents were not a priority group, and structural drivers such as unhealthy food environments were often overlooked in favour of individual-level explanations. In addition, there was a perception that legislating food producers and vendors (such as sugar taxes or advertising regulation) was infeasible, partly due to the political power of manufacturers in the country. Based on this analysis, the authors generated insights into the prerequisites for successful nutrition policy reforms, including the need for policy entrepreneurs and advocacy coalitions to reframe the issue and sustain momentum for reform through political transitions.

### Methods and study designs

In this section, we present an analysis of the range of methodologies and study designs applied, as well as an exploration of the theories and frameworks used. In doing so, we hope to better understand the variety of disciplinary perspectives, analytical approaches, and theoretical conceptions applied in the field of HPA.

The review revealed the wide range of study designs applied in HPA in LMICs. Most notably, we identified case study as the study design for 132 articles (of 629: 21%). Gilson and Raphaely (2008) similarly found that a significant proportion of articles present case studies, but also noted that most had methodological weaknesses, and that very few explicitly apply formal cross-case analysis approaches that could generate insights of wider relevance. We identified 53 studies described as either multi-case studies, comparative case studies, or single case studies with a comparative aspect (such as between sub-units). Within this set are useful examples of rigorously conducted comparative case studies—demonstrating how comparative studies enable explanation (beyond description) (see Box 2). While this analysis cannot gauge the rigour of the full set of case study articles, the number of comparative case studies suggests some development in the field with respect to efforts to learn from in-depth analysis of multiple policy experiences.

Box 2 **Examples of case studies with a comparative element used to enable explanation**

The case studies using comparative analyses to enable explanation include comparisons across countries, communities, or policies.

- Pongcharoensuk *et al*. (2012) compare policy formulation in response to avian and pandemic human influenza policy across three countries (Thailand, Indonesia, and Vietnam) to explain why countries’ policy responses may be similar in one policy arena and different in another. The authors find that, while all three countries developed similar antiviral stockpiling policies, Thailand differed by deciding against a poultry vaccination policy. This is explained by different interpretations of available evidence and by the importance of poultry exports to the country’s economy and the power of large poultry producers to oppose the vaccine policy.
- Smith *et al*. (2014) use a replicative case study design to compare across countries (Bolivia, Malawi, and Nepal) and explain why trajectories of political priority for neonatal mortality differed. Global goals, common across all three country cases, help explain the prioritization of the issue, while domestic factors such as variable domestic research and implementation capacity, the presence (or absence/weakness) of policy entrepreneurs and communities, prioritization of related policies, and political transitions make clear why political priority varied.
- Smith (2014) uses sub-national comparators (South Indian states) to explore how political context influenced maternal health policy. This study purposefully selected one divergent case to identify the conditions that help explain alternative outcomes.
- Amoah (2019) uses sub-national units (two communities in Ghana) to compare the functioning of Community-Based Health Planning Services and explore how differences in social capital affected implementation and functioning of the programme.
- Green *et al*. (2011) apply a comparative analysis of four maternal health policies in three Asian countries, to identify factors hindering policy change processes. The approach shows how factors beyond the policy issue itself, such as resonance between the policy proposal and broader socio-economic development plans, influence policy change.

Beyond case studies, other study designs applied included mixed methods (combining qualitative and quantitative data), stakeholder analysis, realist evaluation, process tracing, and political economy analysis (PEA). PEA and other methods grounded in political sciences offer opportunities to explore the relationship between ‘big P’ politics and political institutions, and health policy change. Box 3 explicates the emergence and application of an analytical framework for PEA within the field.

Box 3 **The emergence of a PEA framework for HPA**

Sparkes *et al*. (2019) present an analytical framework for PEA of health financing reform that draws attention to the politics of six major stakeholder groups relevant to health financing reform, and use the approach to analyse the trajectory of financing reforms in Turkey and Mexico. The framework has since been applied in several studies:

- Khanal *et al*.’s (2023) PEA of the implementation of Nepal’s NHI Programme
- Novignon *et al*.’s (2021) analysis of Ghana’s efforts to achieve UHC through NHI
- Fox and Choi's (2019) analysis combining process tracing with PEA to explain the lack of progress towards NHI reforms in the USA

As an example of the introduction of a new analytical framework to the field, which then enables further research, the introduction of the Sparkes *et al*. framework is evidence of the evolution of the field of HPA in recent years.

Studies using process tracing approaches highlight the value of paying attention to timing and history in analyses of policy processes. The approach uses triangulation across multiple sources and types of data, and a context- and history-sensitive analysis, to reveal processes and establish causality (Smith et al. 2014, Ulriksen and Dadalauri 2016). For example, Smith *et al*. (2014) use this approach to explore how and why political priority for newborn survival has waxed and waned over time, while Lavers (2019) applies a process tracing methodology to the experience of Community Based Health Insurance introduction in Ethiopia to assess the explanatory potential of two theories for explaining health insurance evolution.

Other, more niche study designs identified included ethnographic and anthropological studies, action research, historical approaches, and interpretive methods including discourse analysis. Indeed, although moving away from analyses of policy content is a central facet of HPA, this review reveals some of the ways analysts can use critical or interpretive forms of content analysis to help reveal the power and politics of health policy processes (see Box 4).

Box 4 **The role of critical and interpretive forms of content analysis in revealing power dynamics in policy processes**

The review identified several studies using discourse analysis and related approaches to explore power and politics through an analysis of the content of documentary evidence. Some, such as Barlow and Thow (2021) and Bust et al. (2023), analyse policy-related documentation (such as meeting records or position statements) to demonstrate how powerful actors use discursive techniques to legitimize (or delegitimize) other actors and their interests. Other studies use theory-informed approaches to content analysis to explore a similar range of issues:

- Atuoye *et al*. (2016) use an analytical approach described as ‘content analysis’ to explore rhetoric used by stakeholders in the debate around the Capitation Claims Payment policy for the Ghanaian NHI, revealing the politics underlying pro- and anti-capitation positions. Their analytical approach, informed by Howland *et al*. (2006) and drawing on Lasswell’s policy analytic framework, mapped the symbolic environment in which the policy processes unfolded.
- Bergen *et al*. (2021) describe their analytic approach as a combination of ‘thematic and content analysis’ but apply Bacchi’s ‘what’s the problem represented to be’ approach to maternal, neonatal, and child health in Ethiopia, to explore how health inequity is framed as a policy issue. Their approach allowed exploration of the ways in which current framings of the problem of inequity are incentivized by power dynamics between global funders, national ministries and decision-makers, and implementers and serve to obscure some aspects of the problem, such as global social relations and in-country socio-political realities.

However, while discourse analysis can enable the surfacing of ‘hard-to-see’ power dynamics, it is often used more narrowly, resulting in analyses of content that neglect factors such as which actors contribute to establishing policy discourse and how particular policy discourses gain primacy over others.

#### Theories and frameworks

We briefly surveyed the range of theories and frameworks applied in the included studies and Table 3 presents those most commonly identified. Walt and Gilson’s HPA Triangle model was particularly widely used. This is likely partly a reflection of the framework’s simplicity, which means it can be used by researchers who may be relatively inexperienced in policy analysis, and partly the fact that the framework is not stage-specific and can be usefully applied to a broad range of types of analysis.

**Table 3:** The theories and frameworks most commonly applied in the included studies.

Walt and Gilson’s HPA Triangle
Kingdon’s Multiple Streams Framework
Shiffman and Smith’s Priority Setting
Lipsky’s Street-Level Bureaucracy
Bossert’s decision space
Complexity/CAS
Historical Institutionalism
Hall’s Ideas, Interests, and Institutions
Sabatier’s Advocacy Coalition Framework
Gaventa’s Power Cube
Path Dependency Theory
Veneklasen and Miller Power
Grindle and Thomas’ Political Economy of Reform model
Ulucanlar *et al*.’s Policy Dystopia Model
Stages Heuristic
Bacchi’s What’s the problem represented to be
Kapiriri and Martim’s Priority Setting
Fox and Reich’s UHC Reform Framework

The analysis also revealed a range of significant social science theories and frameworks that are surprisingly rarely applied in the field. These include Bourdieu's theory of practice, Cultural-Historical Activity Theory, Giddens' structuration theory, and Fairclough’s concepts interdiscursivity and recontextualization. There is also evidence of the use of theories and frameworks from the wider policy sciences, such as the Analysis of Determinants of Policy Impact framework, the diffusion of innovations framework, and Ostrom’s theory of polycentric governance. In addition, a few included studies speak to the continued development of the field insofar as they explicitly contribute to the development of HPA theory or testing of existing theory for relevance to modern LMIC contexts (see Box 5). These studies contribute to the ongoing debate in the field regarding the relevance of existing theories to HPA in particular contexts (Powell and Mannion 2022, Gilson and Walt 2023, Whyle et al. 2024).

Box 5 **New HPA theories and papers testing existing theories for applicability to LMIC contexts**

We identified a few papers either introducing new analytical frameworks or testing the applicability of existing theories and frameworks to contemporary LMIC contexts. Sparkes *et al*.’s introduction of a PEA framework is discussed in Box 3. Additionally, we identified a number of papers seeking to test or refine existing frameworks:

- Zheng *et al*. (2010) test whether policy network theory can be usefully applied to health policy processes in China, given particularities of the Chinese political system and find that ‘most, if not all, the theoretical notions developed around policy networks proved fruitful’ in explaining health policy change in China, if applied with sufficient sensitivity to context.
- Aniteye and Mayhew (2013)—mentioned above as a noteworthy example of rigorous implementation research—extend SLB theory by explicitly seeking to account for providers’ values and beliefs and the personal dilemmas that arise when these conflict with policy prescriptions.
- Lavers (2019) traces the process of Community Based Health Insurance introduction in Ethiopia and assess the potential of two theories for explaining health insurance evolution.

### Subject areas studied

In this section, we explore the range of subject areas studied in HPA studies.

The HPA evidence-base covers a diverse array of health and health systems subject areas. Table 4 presents the number of articles on each subject. Note that some articles have more than one main theme, and for some articles, the health or systems subject was indiscernible from the abstract or key words. Maternal and child health was by far the most common subject area, followed by HIV, food and nutrition, and non-communicable diseases. With regard to health systems areas, financing issues comprised the largest set. In this analysis, we distinguished between major financing reforms (National Health Insurance or NHI and Social Health Insurance), user-fee removal and related issues such as exemptions (which accounted for a particularly large number of articles), and other financing issues (such as performance-based financing, provider reimbursement models, and donor aid). Across these three sub-categories, we identified 108 (17%) articles (compared to Gilson and Raphaeli’s 13% across the ‘health financing,’ ‘donor coordination,’ ‘insurance’, and ‘user fees’ categories). Together, articles on financing issues made up 40% of all the articles on health systems subjects. Other prominent health system areas were human resources for health and service delivery issues (including the organization of health services and benefit packages).

**Table 4:** The number of included articles per health and health system subject area.

Health subject area		Health system area	
Maternal and child health	73	Financing (excluding user fee removal)	47
HIV	41	Human resources for health	45
Food and nutrition	35	National/social health insurance	40
Non-communicable diseases (including obesity)	33	Service delivery (organization and benefits)	36
Tobacco control	30	User fee removal	21
Sexual and reproductive health (including termination of pregnancy)	25	Planning and policy	18
Primary healthcare	22	Health systems reform (major reforms excluding national/social health insurance)	14
Marginalized and vulnerable groups (indigenous people, children, adolescents, migrants, etc.)	15	Decentralization	13
Malaria	13	Private sector engagement (including public-private partnerships, public-private mix, and regulation)	9
Mental health (including substance abuse)	13	Public participation	7
Tuberculosis	12	Drug and health commodities (regulation and pricing)	6
Communicable disease (excluding COVID-19, tuberculosis, malaria, and HIV)	10	Governance	6
Oral health	9	Inter-sectoral collaboration	6
Communicable diseases: COVID-19	8		
Integrated community case management of childhood illness	8		
Alcohol regulation	6		
Vaccines	6		
Antimicrobial resistance	5		
Articles on topics with <3 occurrences			20

While it must be recognized that the categorization of articles was iterative (and is fairly subjective)—and that direct comparison to the findings of the prior review is not possible—this analysis does suggest some interesting trends and developments. Some of the categorization differences are themselves reflective of developments in the field. For example, the prior review’s ‘safe motherhood category’ (comprising 7 articles, excluding child health) versus the present review’s ‘maternal and child health’ (comprising 73 articles) is reflective of a development in the broader field in which maternal and child health are recognized as closely linked and services increasingly integrated. Similarly, the categories ‘non-communicable diseases’ and ‘food and nutrition’ in the present review reflect the rise in attention to the social determinants of health.

Gilson and Raphaely suggested that the small number of subject areas with more than 20 articles (the authors identified 6) revealed the fragmented nature of the field. While we agree that there are still many policy topics to which limited attention is paid by policy analysts and which lack robust bodies of HPA evidence, we identified 12 subject areas with more than 20 articles, and argue that a robust body of HPA evidence has emerged around particular topics. For these topics, the number of HPA studies, the depth of analysis within each study, the diversity of theories and perspectives applied to understand the issue, and the cumulative building of the body of knowledge over time allows for the emergence of generalizable lessons about the range of factors that help to explain policy processes for that topic, and more broadly. This is true for bodies of evidence on policy processes for particular health or health systems issues—such as the politics of agenda-setting for maternal and child health (MCH, presented in Box 6) and the importance of accounting for history in analyses of health financing reforms (presented in Box 7)—or a policy change experience in a particular country. As an example of the latter, the HPA evidence-base on the Ghanaian NHI presents a set of papers that together demonstrate the political nature of decision-making around a particular issue, the long-term consequences of particular decisions, the influence of policy frames and counter frames in determining stakeholder positions, and the political challenges experienced in efforts to refine financing policies after their introduction (see Agyepong and Adjei 2008, Seddoh and Akor 2012, Abiiro and McIntyre 2013, Atuoye et al. 2016, Wireko et al. 2020, Abiiro et al. 2021, Novignon et al. 2021). This set of papers is testament to the value of a body of HPA work exploring a specific policy issue over time in a specific setting, together providing a rich and nuanced account of the policy experience unfolding over time.

Box 6 **The robust body of HPA evidence on the politics of agenda-setting for MCH**

A number of these articles use social science theories and frameworks, and often case study methodologies, to explain the politics of agenda-setting for MCH.

- Jat *et al*. (2013) use Kingdon’s multiple streams framework to examine agenda-setting for maternal health in Madhya Pradesh, India.
- Smith (2014) also explores agenda-setting (along with implementation) at the sub-national level in India, drawing on ideas from Kingdon’s multiple streams framework and Sabatier’s advocacy coalition framework, and using a comparative case study approach and process tracing methods.
- Smith *et al*. (2014) compare the prioritization of neonatal mortality across three LIC cases, also using process tracing, but drawing on earlier work on the topic to develop a theory-informed analytical framework (Shiffman 2007).
- Smith *et al*. (2019) use an analytical framework drawing attention to the role of ideas, interests, institutions, and actors to study agenda-setting for maternal survival in Ghana and Tanzania.
- Uzochukwu *et al*. (2020) combine elite theory with Kingdon’s multiple streams framework to explain prioritization of MCH policy in Nigeria.
- Mukuru *et al*. (2021) also use elite theory to explain MCH policy processes in Uganda, finding that policy processes are driven by elite interests.

Taken together, these studies point to the importance of recognizing power, politics, history, and context, using theoretically informed analytical approaches to do so. They also demonstrate the value of case study methodologies—which lend themselves to triangulation across data types and keen attention to context—and, in particular, well-designed comparative case studies, which enable the identification of factors that explain similarities and differences between MCH policy experiences.

Box 7 **HPA on health financing reform and the value of history-sensitive analysis**

A relatively large body of HPA scholarship is emerging on the process of major financing reforms in LMICs, paying particular attention to history, change over time, and path dependency.

- Pisani *et al*. (2017) present a ‘historically rooted’ analysis of UHC reforms in Indonesia from 1945 to 2014 and demonstrate how historical decisions create entrenched bureaucratic interests that constrain change over time, but also how such constraints can be overcome.
- Tangcharoensathien *et al*. (2019) draw on insider observation to explain UHC reform in Thailand over a 40-year period and, like Pisani *et al*., identify factors that contribute to path dependency and factors that enabled change in spite of it.
- Wireko *et al*. (2020) deepen the application of path dependency theory in HPA by applying the concept of self-reinforcing policy feedback, to analyse the transition from the ‘Cash and Carry’ fee for service model (introduced in the 1970s) to the establishment of the NHI System in Ghana in 2003.
- Whyle and Olivier (2023) use a retrospective review to explore health system reform efforts in South Africa since the 1920s and reveal that while historical decisions create entrenched bureaucratic and financial interests that resist change, shared histories also aid pro-reform advocacy.

These papers have relatively loosely defined methods, particularly regarding data analysis approaches, suggesting a possible trade-off between broader analyses of events over a longer timescale and the granularity of data presented. To maintain rigour, many use some form of periodization to explain the dynamics of policy change over time. In addition, these papers tend to draw heavily on theory and frameworks. Together, this set of papers demonstrates the value of a wider time-horizon and history-sensitive analytical approaches, including the ability to illuminate entrenched positions of actors (Pisani et al. 2017, Whyle and Olivier 2023) and the role of bureaucratic politics in entrenching these positions (Tangcharoensathien et al. 2019), to make sense of the political considerations driving decisions at various points in history (Onoka et al. 2015), and to expose the mechanisms by which path dependence is ensured or overcome (Pisani et al. 2017, Wireko et al. 2020).

#### Neglected topics

While robust bodies of work have emerged on some topics, the review also indicates that some health and health systems policy issues remain relatively neglected from a policy analysis perspective. For example, we identified only five articles on policy processes for anti-microbial resistance (AMR). This is an issue of significant and immediate concern, and while the World Health Assembly passed a Resolution on AMR in 1998 (World Health Organization 2001), the earliest paper we identified on the topic was published in 2019. While this limited number of studies may reflect the fact that AMR policies tend to be multi-sectoral, and therefore both more challenging to analyse and not necessarily firmly located within *health* policy analysis, it may also be a reflection of the relatively low priority given to AMR by donors and research funders.

Similarly, we identified relatively few articles on vaccines and immunization programmes (excluding COVID-19 vaccines, which were categorized under COVID-19) and alcohol regulation. Regarding alcohol regulation, with few exceptions (such as Bertscher et al.’s 2018 work on policy formulation processes for alcohol marketing regulation), HPA studies on alcohol policy are either analyses of policy content, or broadly focused on non-communicable diseases policy, rather than alcohol specifically. Given the powerful influence of industry actors in both sectors, there are likely a range of lessons to be drawn from the relatively robust body of work on tobacco control and regulation (see Table 4), and it is worth pursuing further HPA of alcohol control policy processes, including the factors that might keep alcohol regulation off national policy agendas. Hopefully, the recent increase in attention to health taxes more broadly will catalyse further research on this topic.

## Discussion and conclusion

Since 2008, the volume of published HPA in LMICs has grown substantially. This growth is likely attributable, at least in part, to the concerted efforts of individuals and institutions to expand and strengthen the field, including through the development of resources such as the HPA reader (Gilson et al. 2018).

However, while the review shows some progress with respect to the distribution of evidence among countries of different levels of socio-economic development, it also shows that the significant imbalances identified in the 2008 review persist. North Africa and Central Asia remain severely understudied, and more studies still focus on the experiences of lower-middle-income and middle-income countries than those of LICs. Fragile and conflict-affected (FACA) settings are also under-researched. Although a full analysis of FACA states is difficult (because the list of FACAs changes annually) and was beyond the scope of this article, a rough comparison with the 2008 review (based on the World Bank’s current FACA states list) suggests a decline in papers focused on these contexts. In an era of rising global political instability, better understanding policy change dynamics in such contexts will be particularly valuable, and further research in this area is warranted. It must be noted that the exclusion in this study of work in languages other than English constitutes a significant limitation. The results of a more inclusive review may show a more equitable distribution of scholarly literature, and such a review would enable a more nuanced accounting of the state of the field.

Another enduring imbalance is the dominance of authors based in HICs. Despite efforts to strengthen the field of HPA in LMICs, authors based in HIC institutions continue to publish almost half of all papers in the field, and there are particularly few articles first-authored by authors based in LICs. These findings mirror publishing trends for other health topics and fields (see, e.g. Hasnida et al. 2017, Schneider and Maleka 2018, Bhakuni and Abimbola 2021, The Lancet Global Health 2021). In addition, the fact that only a small percentage of HPA authors publish more than one article suggests that many who engage in the field lack the institutional or financial support needed to sustain their research. Addressing this requires deliberate and steady investment in LMIC institutions. Otherwise, it is likely that the problematic maldistribution of power in the production of HPA knowledge—evidenced by the difference between the number of studies on LMIC experiences and the number of authors based in LMICs—will persist. Efforts such as the Health Policy Analysis Mentorship Programme, a collaboration between the Alliance for Health Policy and Systems Research and the University of Cape Town, which provided training and financial support to over 20 LMIC-based doctoral students, have contributed to the emergence of a cohort of LMIC-based HPA researchers (Gilson et al. 2021, AHPSR 2025). However, far more needs to be done to establish a more broad-based HPA community within and across LMICs. Given the inherent challenges of development assistance, including for research, it is important that such endeavours are funded using domestic and regional resources.

Beyond the volume of studies and their authorship, we argue that this review shows substantive development in the field since 2008. One indicator of this development is the increased number of studies using core HPA concepts, such as the language of the policy process phases model, to explain and structure their analyses.

Another indicator of progress is the increased depth of theoretical engagement in published literature. Gilson and Raphaely’s (2008) review highlighted the limited use of theory in HPA as a particular weakness of the HPA evidence-base, and called for deeper engagement with existing policy analysis theory to enable explanation and generalization. In this respect, we suggest that the application of a wide range of established theories and frameworks from social and political sciences is indicative of some development in the field. In addition, alongside continued application of core HPA frameworks, we note efforts to test and refine existing theory based on empirical evidence of LMIC experiences. Nonetheless, many well-established social science theories that hold significant potential value for HPA are still very rarely applied, and there remains much scope to expand and deepen the use of theory in HPA. In addition, there is a need to continue to test, refine and adapt existing theories and frameworks based on evidence of contemporary LMIC policy processes. Such adaptation would likely enhance the real-world applicability of HPA to policy and practice in these settings, and, in turn, make the case for the value of HPA and stimulate investment from domestic governments and research funders.

We also note maturation in the field with regard to HPA on implementation, and, particularly, the use of theory therein. Gilson and Raphaely (2008) found that implementation was the most frequently studied phase of the policy process, but argued that most HPA on implementation in LMICs failed to utilize much of the available implementation theory, and tended to neglect consideration of software factors such as organizational culture and power. Our findings mirror those of the earlier review with respect to the number of studies on implementation. While this does not on its own suggest that the number of rigorous studies paying close attention to implementation processes is sufficient, our review revealed some encouraging examples of HPA on implementation. These include studies that draw on social science theory to expose how software factors such as power, trust, and values shape implementation, and studies that explicitly seek to account for the dynamic relationship between implementation processes and the complex adaptive health systems within which they unfold. Nonetheless, deeper understanding of how implementation interacts with policy formulation and dissemination processes, actor power and politics, health system dynamics, and contextual realities remains warranted.

Another indicator of maturation of the field is the number of comparative studies. Gilson and Raphaely (2008) noted few studies with a comparative element, which, they argued, hinders the development of explanatory theories and generalizable lessons. The present review suggests progress in this regard, particularly with respect to the number of studies using comparative case study methods—which can explain the causal influence of various factors, offer substantive explanations for why a particular policy process unfolded as it did, generate lessons of wider relevance, and enable cross-contextual learning. We also note progress with regard to less commonly applied methodological approaches, such as PEA (for which we note Sparkes *et al*.’s PEA framework, since applied in several of the studies reviewed) and process tracing methods, which allow for politics- and history-sensitive analyses. While the number of studies using these approaches remains small, there is evidence of methodological and theoretical development, with subsequent studies developing from, and building on, the methodologies of earlier work.

While these methodological and conceptual developments are encouraging, it is still the case, as we found through the screening process for this study, that many studies labelled as ‘health policy analysis’ are in fact evaluations of policy outcomes or analyses of policy content, without consideration of process. During screening, we excluded over 400 policy assessment or evaluation articles, and over 150 articles that were purely descriptive and neglected any consideration of process (including analyses of policy content only). The preponderance of purely descriptive studies of policy processes, noted as a weakness by Gilson and Raphaely (2008), clearly persists. In addition, our review suggests that ‘health policy analysis’ is still often taken to include analyses of policy content or evaluations of policy impact, particularly in research funded by development agencies.

That being said, while we excluded analyses of policy content that neglect issues of power and process, our analysis does reveal the value of interpretive and theory-informed content analysis in exhuming power and process factors. This is in keeping with Gilson and Raphaely’s call for more HPA using analytic approaches that expose the power of rhetoric, discourse, framing, and narrative in policy processes. We concur and suggest that studies using theory-informed forms of content analysis show how apparently neutral and uncontested framings can obscure contestation and protect the interests of particular actors, and how powerful actors use discursive and rhetorical techniques to legitimize or marginalize others’ concerns and influence policy processes. Such work demonstrates that some forms of content analysis, when attuned to power and process, can be an important form of HPA.

Another way in which the review suggests development in the field is the number of topical areas for which robust bodies of evidence are emerging. The 2008 review called for the development of robust areas of work through sets of studies on a particular topic (either within, or between countries) that build on one another to deepen understanding. We found several examples of this including sets of articles on a particular topic across contexts, such as the politics of agenda-setting for MCH, or on a particular policy experience in a single setting but captured in a series of articles analysing various facets or periods of the policy process, such as for the NHI policy process in Ghana. There is significant value in these robust areas of work, which allow for cumulative learning and deeper understanding, accounting for emerging evidence from a range of methodological and theoretical perspectives.

However, we also note that some important topics, where HPA research would be of significant value, remain neglected—such as policy processes for anti-microbial resistance initiatives, immunization programmes, and alcohol regulation. Similarly, the study of processes seeking to bring about policy change, noted as an area of particularly limited research by Gilson and Raphaely (2008), remains neglected, with few studies analysing advocacy efforts and few examples of prospective HPA. The small body of work in this area demonstrates its potential to generate real-world insights of direct relevance to decision-makers and advocates, and further work—either in the form of more prospective HPA studies of policy advocacy initiatives, or analyses demonstrating the applied value of such approaches—is strongly encouraged.

Taken together, our findings point to a field that has grown in scale, scope and analytic sophistication, but which still requires further, deliberate nurturing. Here, we draw on the foregoing analysis to offer suggestions as to how to sustain momentum in the development of the field of HPA:

1. Support institutions in LMICs, particularly in under-studied contexts such as North Africa, Central Asia, and FACA states, and devise institutional arrangements, including funding, to support researchers conducting HPA in their own contexts. This will help to ensure that HPA researchers are able to find institutional support for their research and build careers while continuing to live in their home countries, or country of expertise. There is also a need to deepen and extend existing institutional capacity in LMICs, ensuring that this capacity is sustained and able to anchor the development of the field in more emerging contexts.
2. Strengthen mechanisms to prevent parachute research and HIC dominance in studies of LMIC contexts. This would include publishing practices that require author reflexivity statements prior to publication and empower editors to question submissions in which HIC authors lead research on LMIC experiences—an approach some journals in the field are already using. Expanding domestic and regional funding for HPA and ensuring genuine partnership and power-sharing in cross-context research collaborations will also be key to supporting more equitable knowledge production in the field.
3. Encourage and enable analytical HPA that reveals causal factors and generates lessons of wider relevance including by funding longer-term studies and cross-country research partnerships.
4. Deepen theoretical engagement, including more work drawing on social science and political science theory, and further efforts to translate social science theories for analysts trained in biomedical or other ‘hard science’ disciplines, for example, by being more explicit about the value of these theories to answering core HPA questions.
5. Demonstrate the applied value of HPA, by directly, and timeously, addressing real-world policy challenges and showing how HPA contributes to and enables progressive policy change. Embedded research approaches, co-creation with policymakers, and sustained forums for policymaker-researcher engagement—such as journal clubs or think tanks—can help achieve this, provided they are initiated by policy-makers themselves and supported by local investment.
6. Strengthen prospective HPA, by identifying barriers to such work and creating funding and institutional conditions that support it.

HPA in LMICs has expanded and matured considerably in recent years and can no longer be considered ‘in its infancy’. Nonetheless, its continued relevance depends on its ability not only to document policy processes, but to make sense of them, learn from them, and shape them. This requires theoretically robust, contextually sensitive analysis that strengthens both scholarship and policy practice. Purposeful efforts on the part of researchers, funders, and institutions are still needed to strengthen and diversify the field, to ensure that HPA realizes its full potential and contributes to progressive policy change. Sustained and deepened HPA capacity across country settings is the foundation for this work.

## Supplementary Material

czag079_Supplementary_Data

## Data Availability

As this is a review of published HPA scholarship, the data used in this analysis is entirely in the public domain.

## Reflexivity statement

All three authors are experienced health policy analysts, who have themselves conducted relevant studies and taught in this area. The first author (E.B.W.) is a South African researcher who has lived, studied, and worked in South Africa. The second author (L.G.) holds a joint appointment with a UK university, but has lived and worked in South Africa for several decades and has substantial experience in a wider range of LMIC contexts. The third author (Z.C.S.) is an Indian national, currently working in Geneva and supporting research across settings. All three authors are deeply embedded in the global health policy and systems research community and have extensive familiarity with systems of research and knowledge production in LMIC and high-income contexts.
