# Supplementary material for: Developments in the field of health policy analysis in low- and middle-income countries: a review of published literature 2008–2023
Source: Health Policy Plan. 2026 Jun 25;41(8):1473–92. doi: 10.1093/heapol/czag079 (PMC13586625; doi:10.1093/heapol/czag079)
Supplement: Supplementary file 1 [file czag079_supplementary_data.zip › Supplementary data 1_Methodological details.docx]

# Supplementary data 1: Methodological details

## Rationale for deviating from original search strategy

While the present review is, in some ways, a follow-up to the 2008 review, a somewhat different methodological approach was taken. This was necessary due to the massive expansion in academic publishing. Academic publishing (i.e. the number of academic articles published per year) has been rising exponentially since about the 1950s (Thelwall & Sud 2022), and has continued to do so since 2008. For this reason, a search strategy that would have returned a manageable number of results in 2008, now has to be further refined. In this case, at the time of the study, running a similar search to that used by Gilson and Raphaely for the period 2008-2024 in the International Bibliography of Social Sciences gives 11 586 results, and in Pubmed gives 12 477 results (compared to the ‘several thousand’ returned by Gilson and Raphaely’s search).

Like Gilson and Raphaely, our intention was not to systematically review all evidence on policy change processes in LMICs, but rather to assess the development of the field since the publication of that review. As such, given the need to develop a more refined search strategy than that used by Gilson and Raphaely, we used different data collection approaches to identify empirical studies experiences of health policy change in LMICs only.

## Policy process terms

"implementation" OR "implementer" OR "implementers" OR "political" OR "politically" OR "politics" OR "politic" OR "agenda-setting" OR "agenda setting" OR "power" OR "interest" OR "interests" OR "discourse" OR "discourses" OR "discursive" OR "health advocacy" OR "construct" OR "constructs" OR "construction" OR "policy-making" OR "policy making" OR "policy-analysis" OR "policy analysis" OR "power" OR "actor" OR "actors" OR "values" OR "ideas" OR "ideologies" OR "ideology" OR "ideological" OR "ideologically" OR "process" OR "processes" OR "context" OR "contexts" OR "contextual" OR "contextualization" OR "contextualize" OR "contextualized" OR "contextualizes" OR "contextualizing" OR "theories" OR "theory" OR "social" OR "sociological" OR "sociologic" OR "sociologically" OR "historic" OR "historical" OR "historically" OR "historicity" OR "history" OR "histories" OR "prioritisation" OR "prioritisations" OR "prioritise" OR "prioritised" OR "prioritises" OR "prioritising" OR "prioritization" OR "prioritizations" OR "prioritize" OR "prioritized" OR "prioritizes" OR "prioritizing" OR "policy agenda" OR "priority-setting" OR "decision-making" OR "decision making" OR "policy formulation" OR "policy planning" OR "stakeholder analysis" OR “street level bureaucrats” OR “street-level bureaucrats” OR “street level bureaucracy” OR “street-level bureaucracy”

## MeSH major topics list

"Policy Making" OR "Health Policy" OR "Health Policy/economics" OR "Health Policy/history" OR "Health Policy/legislation and jurisprudence" OR "Health Policy/trends" OR "Financial Management" OR "Financial Management/history" OR "Financial Management/legislation and jurisprudence" OR "Financial Management/organization and administration" OR "Financial Management/standards" OR "Health Services Research" OR "Health Services Research/history" OR "Health Services Research/legislation and jurisprudence" OR "Health Services Research/organization and administration" OR "Health Services Research/standards" OR "Health Services Research/trends" OR "Decision Making, Organizational" OR "Delivery of Health Care" OR "Delivery of Health Care/ethnology" OR "Delivery of Health Care/history" OR "Delivery of Health Care/legislation and jurisprudence" OR "Delivery of Health Care/organization and administration" OR "Delivery of Health Care/trends" OR "Delivery of Health Care, Integrated" OR "Delivery of Health Care, Integrated/history" OR "Delivery of Health Care, Integrated/legislation and jurisprudence" OR "Delivery of Health Care, Integrated/organization and administration" OR "Delivery of Health Care, Integrated/statistics and numerical data" OR "Delivery of Health Care, Integrated/trends" OR "Health Care Reform" OR "Health Care Reform/history" OR "Health Care Reform/legislation and jurisprudence" OR "Health Care Reform/organization and administration" OR "Health Care Reform/trends" OR "Health Planning" OR "Health Planning/history" OR "Health Planning/legislation and jurisprudence" OR "Health Planning/organization and administration" OR "Health Planning/trends" OR "National Health Programs" OR "National Health Programs/history" OR "National Health Programs/legislation and jurisprudence" OR "National Health Programs/organization and administration" OR "National Health Programs/trends" OR "Organizational Culture" OR "Politics" OR "Power, Psychological"[Mesh:NoExp] OR "Public Policy" OR "Public Policy/history" OR "Public Policy/legislation and jurisprudence" OR "Public Policy/trends" OR "Nutrition Policy" OR "Nutrition Policy/history" OR "Nutrition Policy/legislation and jurisprudence" OR "Nutrition Policy/trends" OR "Tobacco Control" OR "Tobacco Control/history" OR "Tobacco Control/legislation and jurisprudence" OR "Tobacco Control/trends" OR "International Health Regulations" OR "International Health Regulations/history" OR "International Health Regulations/organization and administration" OR "International Health Regulations/trends" OR "Public Sector" OR "Public Sector/history" OR "Public Sector/legislation and jurisprudence" OR "Public Sector/organization and administration" OR "Public Sector/trends" OR "Government Programs" OR "Government Programs/history" OR "Government Programs/legislation and jurisprudence" OR "Government Programs/organization and administration" OR "Government Programs/supply and distribution" OR "Government Programs/trends" OR "Health Care Rationing" OR "Health Care Rationing/history" OR "Health Care Rationing/legislation and jurisprudence" OR "Health Care Rationing/organization and administration" OR "Health Care Rationing/trends" OR "Health Equity" OR "Health Equity/history" OR "Health Equity/legislation and jurisprudence" OR "Health Equity/organization and administration" OR "Health Equity/trends" OR "Health Plan Implementation" OR "Health Plan Implementation/history" OR "Health Plan Implementation/legislation and jurisprudence" OR "Health Plan Implementation/organization and administration" OR "Health Plan Implementation/trends" OR "Health Planning Guidelines" OR "Health Planning Technical Assistance" OR "Health Planning Technical Assistance/history" OR "Health Planning Technical Assistance/legislation and jurisprudence" OR "Health Planning Technical Assistance/organization and administration" OR "Health Planning Technical Assistance/trends" OR "Regional Health Planning" OR "Regional Health Planning/history" OR "Regional Health Planning/legislation and jurisprudence" OR "Regional Health Planning/organization and administration" OR "Regional Health Planning/trends" OR "Certificate of Need" OR "Certificate of Need/history" OR "Certificate of Need/legislation and jurisprudence" OR "Certificate of Need/organization and administration" OR "Certificate of Need/trends" OR "Community Health Planning" OR "Community Health Planning/history" OR "Community Health Planning/legislation and jurisprudence" OR "Community Health Planning/organization and administration" OR "Community Health Planning/trends" OR "Health Systems Plans" OR "Health Systems Plans/history" OR "Health Systems Plans/legislation and jurisprudence" OR "Health Systems Plans/organization and administration" OR "Health Systems Plans/trends"

## Search strategy for AfricaWide and SciELO

These searches combined two strategies as follows:

1. Purposive search combined
   1.1. “health policy analysis” (any field), and
   1.2. LMIC country names (title/abstract)
2. Slightly broader purposive search combined
   2.1. Policy (keyword/title)
   2.2. Health (abstract)
   2.3. Policy process terms based on Gilson and Raphaely review^[1]^
   2.4. LMIC country names (abstract)

## Quick inclusion criteria

The ‘quick inclusion’ phrases included:

- Titles of key HPA texts (including in reference lists),
- Names of authors closely associated with HPA, and
- Names of, and key phrases from, foundational HPA theories.

## Quick exclusion criteria

Items were scanned for exclusion if they met any of the following criteria:

- Commentaries and editorials (items with ‘comment’ or ‘commentary’ in ‘Any Field’^[2]^
- Technical economics papers (Health Technology Assessment, cost-effectiveness analysis or similar in Any Field)
- Purely technical/quantitative analyses (modelling, statistical or similar in Title)
- Papers reporting the views/perspectives of policy-makers or users on a policy without connecting these to an analysis of the policy process (papers with ‘perspective’, ‘attitude’ and synonyms in title).
- Policy assessments or evaluations rather than process analyses (assessment, evaluation, or similar in title)
- Papers focusing on producing or translating evidence for policy, i.e. to make policy recommendations, without connecting these to an analysis of the policy process (‘policy’ included in title or abstract only in the context of recommendations or evidence for policy)
- Study protocols (protocol in title)

## References

Thelwall M, Sud P. 2022. Scopus 1900–2020: Growth in articles, abstracts, countries, fields, and journals. *Quantitative Science Studies*, **3**: 37-50.

1. analysis OR implementation OR politics OR “agenda setting” OR “agenda-setting” OR power OR “policy actors” OR interests OR “policy discourse” OR “policy formulation” OR “policy development”
2. ‘Any Field’ includes all citation information (including abstract) but excludes the full text
