# Supplementary material for: Developments in the field of health policy analysis in low- and middle-income countries: a review of published literature 2008–2023
Source: Health Policy Plan. 2026 Jun 25;41(8):1473–92. doi: 10.1093/heapol/czag079 (PMC13586625; doi:10.1093/heapol/czag079)
Supplement: Supplementary file 1 [file czag079_supplementary_data.zip › Supplementary data 2.docx]

# Supplementary data 2: Number of studies per country

| **Country of focus** | **No. of articles** |
| --- | --- |
| South Africa | 61 |
| India | 49 |
| Ghana | 44 |
| Brazil | 40 |
| Iran | 40 |
| Kenya | 39 |
| Uganda | 37 |
| Nigeria | 33 |
| China | 32 |
| Tanzania | 26 |
| Malawi | 25 |
| Ethiopia | 23 |
| Thailand | 23 |
| Burkina Faso | 22 |
| Vietnam | 20 |
| Mexico | 19 |
| Indonesia | 17 |
| Nepal | 14 |
| Cambodia | 13 |
| Cameroon | 13 |
| Zambia | 13 |
| Philippines | 13 |
| Bangladesh | 12 |
| Pakistan | 11 |
| Niger | 9 |
| Turkey | 9 |
| Mali | 8 |
| Mozambique | 8 |
| Bolivia | 7 |
| Colombia | 6 |
| Fiji | 6 |
| Lebanon | 6 |
| Liberia | 6 |
| Rwanda | 6 |
| Ukraine | 6 |
| Benin | 5 |
| Peru | 5 |
| Sudan | 5 |
| Timor-Leste | 5 |
| Zimbabwe | 5 |
| Afghanistan | 4 |
| Burundi | 4 |
| Ecuador | 4 |
| Georgia | 4 |
| Lao | 4 |
| Malaysia | 4 |
| Sierra Leone | 4 |
| Sri Lanka | 4 |
| Togo | 4 |
| Cabo Verde | 3 |
| Guinea | 3 |
| Mongolia | 3 |
| Myanmar | 3 |
| Papua New Guinea | 3 |
| Senegal | 3 |
| Tunisia | 3 |
| Argentina | 2 |
| Chad | 2 |
| Costa Rica | 2 |
| Egypt | 2 |
| Macedonia | 2 |
| Moldova | 2 |
| Serbia | 2 |
| Vanuatu | 2 |
| Solomon Islands | 2 |
| Albania | 1 |
| Algeria | 1 |
| Angola | 1 |
| Bosnia and Herzegovina | 1 |
| Botswana | 1 |
| Democratic Republic of Congo | 1 |
| Dominican Republic | 1 |
| El Salvador | 1 |
| Guatemala | 1 |
| Iraq | 1 |
| Jordan | 1 |
| Kyrgyzstan | 1 |
| Lesotho | 1 |
| Madagascar | 1 |
| Mauritania | 1 |
| Mauritius | 1 |
| Montenegro | 1 |
| Morrocco | 1 |
| Nicaragua | 1 |
| North Korea (DPRK) | 1 |
| North Macedonia | 1 |
| Paraguay | 1 |
| Samoa | 1 |
| Syria | 1 |
| The Gambia | 1 |
| Venezuela | 1 |
| Yemen | 1 |
